# Amino acid‐specific δ^15^N trophic enrichment factors in fish fed with formulated diets varying in protein quantity and quality

**DOI:** 10.1002/ece3.4295

**Published:** 2018-07-30

**Authors:** M. Teresa Nuche‐Pascual, Juan Pablo Lazo, Rocío I. Ruiz‐Cooley, Sharon Z. Herzka

**Affiliations:** ^1^ Departamento de Oceanografía Biológica Centro de Investigación Científica y de Educación Superior de Ensenada (CICESE) Ensenada México; ^2^ Departamento de Acuacultura CICESE Ensenada México; ^3^ Moss Landing Marine Labs San Jose State University Moss Landing California

**Keywords:** carnivorous fish, isotopic fractionation, liver, muscle, nitrogen, nutrient requirement

## Abstract

Compound‐specific isotope analysis (CSIA) of amino acids (AAs) in consumer tissues is a developing technique with wide‐ranging applications for identifying nitrogen (N) sources and estimating animal trophic level. Controlled experiments are essential for determining which dietary conditions influence variability in N stable isotopes (δ^15^N) trophic enrichment factors in bulk tissue (TEF_bulk_) and AAs (TEF_AA_). To date, however, studies have not independently evaluated the effect of protein quantity and quality (digestibility) on TEFs, complicating the application of AA‐δ^15^N values for estimating trophic levels. We conducted a 98‐d feeding experiment using five formulated isoenergetic feeds prepared with a high‐quality protein source to evaluate the effect of protein quantity and quality on TEFs of liver and muscle tissues of juvenile Pacific yellowtail (*Seriola lalandi*), a carnivorous fish species. We decreased protein digestibility using well‐established protocols that do not change AA profiles. Growth rates were higher in diets with higher protein content, and isotopic equilibrium was reached for both fish tissues and all treatments. Protein quantity and quality influenced isotope discrimination depending on tissue type and AA. In liver tissue, bulk TEFs showed a limited but significant relationship with protein quality, but did not differ with protein quantity or quality in muscle. None of the pre‐established source AAs (Lys, Met, Phe, and Gly) TEFs varied significantly with protein quantity or quality in liver tissue. However, in muscle tissue, TEF_Phe_ increased significantly with protein content and decreased in response to reduced digestibility, indicating it may not serve as proxy for baseline isotopic values used to calculate trophic level. Among trophic AAs, TEF_Leu_ decreased significantly with increasing protein quantity in liver tissue, while both Leu and Ile TEFs decreased with lower protein digestibility in muscle tissue. Our results indicate that CSIA‐AA in liver tissue provides more robust source and trophic AA‐δ^15^N values than in muscle.

## 1 INTRODUCTION

Tracing organic material and energy fluxes through food webs is important for determining the functional role of species within an ecosystem. The nitrogen stable isotope ratios (δ^15^N) of bulk consumer tissues have served as powerful natural tracer to infer nutrient sources, characterize animal dietary composition, estimate trophic level, and reconstruct food web structure (Peterson & Fry, 1987). The differences in δ^15^N values between a consumer and its diet_,_ also known as the trophic enrichment factor (TEF), were believed to be relatively constant across food webs and are essential for estimating trophic position (TP) (DeNiro & Epstein, 1981; Minigawa & Wada, 1984). The TEF in bulk tissue (TEF_bulk_) ranges from 2.5 to 5‰ for most soft tissues (reviewed by Vanderklift & Ponsard, 2003; McCutchan, Lewis, Kendall, & McGrath, 2003) and varies depending on diet type (Vander Zanden & Rasmussen, 2001), protein quality (Florin, Felicetti, & Robbins, 2011; Robbins, Felicetti, & Sponheimer, 2005), tissue type (Hobson & Clark, 1992; Malpica‐Cruz, Herzka, Sosa‐Nishizaki, & Lazo, 2012), taxa, and the mode of nitrogen excretion (McCutchan et al., 2003). Because TEF_bulk_ values are incorporated into isotope mixing models to elucidate trophic relationships and food web structure, the use of imprecise TEF_bulk_ values would lead to inaccurate estimates of both TP and the contribution of food sources to tissue production (Phillips, 2012; Post, 2002). Estimating TP requires characterization of the isotopic baseline by measuring the isotopic composition of primary producers (or primary consumers as their proxy) (Cabana & Rasmussen, 1996; Post, 2002). Determination of the δ^15^N_baseline_ is difficult due to high temporal and spatial variability in primary producer isotopic ratios, as well as the temporal uncoupling between source isotope ratios and those integrated by higher level consumers (McMahon, Hamady, & Thorrold, 2013; Popp et al., 2007; Post, 2002).

Compound‐specific isotope analysis (CSIA) of amino acids (AAs) is a developing complementary technique with the potential for reducing the limitations of N stable isotope analysis (SIA) on bulk tissue for estimating TP (e.g., Chikaraishi et al., 2009; McClelland & Montoya, 2002; Ohkouchi et al., 2017; Popp et al., 2007). Some AA δ^15^N values quantified from animal tissues reflect baseline isotope ratios and others consumer trophic level. Currently, source AAs include phenylalanine (Phe), methionine (Met), and lysine (Lys). These AAs presumably reflect primary producer values due to low isotopic discrimination with each trophic step (Popp et al., 2007). In contrast, trophic AAs such as glutamic acid (Glu), aspartic acid (Asp), alanine (Ala), isoleucine (Ile), leucine (Leu), proline (Pro), valine (Val) show large isotopic discrimination with each trophic step. Serine (Ser), threonine (Thr), and glycine (Gly) were initially considered source AAs, but they can exhibit variable and high isotopic fractionation in high trophic level consumers, and do not fit strictly into the source category (Germain, Koch, Harvey, & McCarthy, 2013; McCarthy, Benner, Lee, & Fogel, 2007; McMahon & McCarthy, 2016). N isotope discrimination associated with source AAs (minimal) and trophic AAs (large) has been attributed to whether transamination involves cleavage of a C–N bond (Chikaraishi, Kashiyama, Ogawa, Kitazato, & Ohkouchi, 2007; Chikaraishi et al., 2009). However, isotopic discrimination can also occur during deamination, and both essential AAs (EAA; those that cannot be synthesized de novo by a heterotroph) and nonessential AAs (NEAA) can serve as energy sources producing substrates involved in enzymatic chemical reactions (O'Connell, 2017). A more integrative understanding of the biochemical conditions and processes that discriminate nitrogen isotopes is required. O'Connell (2017) specifies that N isotope discrimination should be considered as the result of an AA transamination, deamination, and the exchange of amino groups within the active N pool.

The difference in TEF_AA_ between a trophic and a source AAs is used to estimate TP, and this difference (e.g., TEF_Glu_ – TEF_Phe_ = 7.6‰ for the canonical AAs) was initially assumed to be constant across species, tissues, and trophic levels from all ecosystems (e.g., Chikaraishi et al., 2009; Popp et al., 2007). Meta‐analyses of AA isotopic fractionation indicate that trophic AAs TEFs exhibit high variability between taxa due to differences in diet composition, taxa, and mode of nitrogen excretion (McMahon & McCarthy, 2016; Nielsen, Popp, & Winder, 2015). Source AAs TEFs can also vary substantially (Steffan et al., 2013; McMahon & McCarthy, 2016 and references therein, O'Connell, 2017). For example, Nakashita et al. (2011) measured blood δ^15^N values of Phe and Glu of long‐term captive black bears (*Ursus thibatanus*) and wild black bears fed with known diets, and found differences of up to 4.6 and 8.5‰ in TEF estimates, respectively. Taxon‐specific empirical estimates of TEFs that evaluate the role of specific dietary protein attributes are necessary. Furthermore, the TPs of marine mammals and other high trophic level predators have been underestimated (e.g., McMahon & McCarthy, 2016; Nielsen et al., 2015) when using CSIA‐AA δ^15^N values and applying the “universal” TEF proposed by Chikaraishi et al. (2009); these results highlighting the need for taxon and TP‐specific TEF estimates.

Two of the main factors influencing the variability in TEFs bulk and AA are quantity and quality of dietary protein (Martínez del Río, Wolf, Carleton, & Gannes, 2009; McMahon, Thorrold, Elsdon, & McCarthy, 2015; Nielsen et al., 2015). Protein is a primary body constituent and an energy substrate. Protein requirements, that is, the minimum amount of protein needed to maximize growth (Dacosta‐Calheiros, Arnason, & Bjornsdottir, 2003), are determined by the EAA requirements of a given species. Protein accretion is a determinant of biomass gain and utilization of AAs, and varies due to endogenous (e.g., life stage) and exogenous (e.g., diet) factors. Martínez del Río and Wolf (2005) made three predictions regarding the relationship between food protein and bulk tissue isotope discrimination: (a) TEF_bulk_ should increase with dietary protein content given that excess dietary protein is catabolized and used as an energy substrate and hence excreted in urine depleted in ^15^N, (b) TEF_bulk_ should decrease with higher protein quality due to the increase in protein intake to meet energy and protein requirements and thus higher AA catabolism, and (c) TEF_bulk_ should decrease with the efficiency of N deposition due to reduced protein catabolism. Experimental studies on fish and other taxa are inconsistent or contradictory regarding the relationship between TEF_bulk_ or TEF_AA_ and protein quality (see review by Martínez del Río et al., 2009; McMahon & McCarthy, 2016). Early studies on CSIA‐AA analyzed the effect of protein quantity on TEF_AA_ dynamics using both wild‐caught and captive specimens of various taxa (e.g., Bradley, Madigan, Block, & Popp, 2014; Chikaraishi et al., 2007, 2009; McClelland & Montoya, 2002; McMahon, Polito, Abel, McCarthy, & Thorrold, 2015; McMahon, Thorrold, et al., 2015). As it has been recognized for SIA in bulk tissues (McCutchan et al., 2003; Vanderklift & Ponsard, 2003), recent studies using CSIA‐AA indicate that diet quality can account for the reported variation in TEF_AA_ between taxonomic groups and trophic levels (Chikaraishi, Steffan, Takano, & Ohkouchi, 2015; Ohkouchi et al., 2017). Feeds with the same protein quantity that overlook variability in protein sources can show pronounced differences in protein quality (McGoogan & Reigh, 1996) due to variations in protein digestibility and AA profile (Masumoto, Ruchimat, Ito, Hosokawa, & Shimeno, 1996). Digestibility is the term used to assess the availability of nutrients to the fish. The term refers to the process of digestion and absorption of nutrients in the digestive system of the organism. Digestion refers to the process of solubilization and hydrolization of nutrient polymers (proteins) into their monomers (amino acids) for latter absorption. Not all proteins are easily digested by fishes; in particular plant proteins have typically low digestibility (see NRC, 2011). For these reasons, independently elucidating the effect of protein quantity and quality within specific taxa will provide the foundation for robust comparisons with other groups with different physiological characteristics.

In fishes, some studies have shown that protein quantity is positively related to TEF_bulk_ (Focken, 2001; Kelly & Martínez del Río, 2010), while others indicate a negative significant relationship (Barnes, Sweeting, Jennings, Barry, & Polunin, 2007; Martín‐Pérez et al., 2013). Regarding CSIA‐AA, an omnivorous fish fed with a low‐protein plant‐based diets resulted in very high δ^15^N TEFs of trophic AAs in comparison with those fed with diets containing animal protein and higher content (McMahon, Thorrold, et al., 2015). Therefore, carnivorous and omnivorous fish fed with vegetable‐based diets with very‐low‐protein content may yield ecologically unrealistic TEFs that should not be applied to wild fish that feed at high trophic levels.

To date, the number of studies investigating the underlying variability in TEF_AA_ is lower than that conducted for TEF_bulk_. Early studies on CSIA‐AA analyzed the effect of protein quantity on TEF_AA_ dynamics using both wild‐caught and captive specimens of various taxa (e.g., Bradley et al., 2014; Chikaraishi et al., 2007, 2009; McClelland & Montoya, 2002; McMahon, Polito, et al., 2015; McMahon, Thorrold, et al., 2015), and only the most recent studies indicate that diet quality influences TEF_AA_ (Chikaraishi et al., 2015; McMahon, Thorrold, et al., 2015). However, studies that report TEF_AA_ estimates based on multiple food sources covaried protein quantity and quality (Table 1), making it impossible to separate the effect of protein quality from protein quantity on TEF variability.

**Table 1:** Summary of studies that examined the effect of dietary protein quantity and quality on TEF_bulk_ and TEF_AA_ in fish. Experiments in which fish were fed a single diet are included for comparative purposes

TEF	Species	Tissue	Protein source in diet	Covary protein quantity and quality	Covary AA profile and digestibility	WR min–max	Reached equilibrium^a^	Reference
TEF_bulk_	*Oreochromis niloticus* (Nile tilapia)	Muscle	Fish meal, wheat gluten, and soybean concentrate	No: only vary protein quantity	No	1.3–2.1	No	Focken (2001)
TEF_bulk_	*Oreochromis niloticus* (Nile tilapia)	Whole body	Two diets: Wheat gluten + EAA Fish meal + wheat meal	Yes	Yes	1.0–3.0	Probably only in fish with highest biomass gain	Gaye‐Siessegger, Focken, and Abel (2003)
TEF_bulk_	*Ciprinus carpio* (Carp)	Whole body	Fish meal + wheat meal‐based commercial diet	No: only varied protein quantity	No	1.0–5.4	Probably only in fish with highest biomass gain	Gaye‐Siessegger, Focken, and Muetzel (2004)
TEF_bulk_	*Oreochromis niloticus* (Nile tilapia)	Whole body	Wheat gluten + synthetic AA	No: only varied protein quantity	ND	1.3	No	Gaye‐Siessegger, Focken, and Abel (2004)
TEF_bulk_	*Dicentrarchus labrax* (European sea bass)	Muscle	Sandeels (non‐formulated diet)	No: only varied protein quantity	N/A	5.8–7.7	Yes	Barnes et al. (2007)
TEF_bulk_	*Oreochromis niloticus* (Nile tilapia)	Whole body	Three diets: EAA + NEAA EAA + AA precursor EAA + glutamate	No: only varied protein quantity	Yes	0.8–1.1	No	Gaye‐Siessegger et al. (2007)
TEF_bulk_	*Oncorhynchus mykiss* (Rainbow trout) *Sparus aurata* (Gilthead sea bream)	Liver, muscle, intestine + perivisceral fat	Fish meal, corn gluten meal, wheat gluten, extruded peas, rapeseed meal soybean meal, extruded whole wheat	Yes	Yes	5.8–7.4	Yes	Beltrán et al. (2009)
TEF_bulk_	*Oreochromis niloticus* (Nile tilapia)	Muscle	Casein	No: only varied protein quantity	No	3.0	Probably	Kelly and Martínez del Río (2010)
TEF_bulk_	*Sparus aurata* (Gilthead sea bream)	Muscle	Fish meal, wheat gluten, and soybean concentrate	No: only varied protein quantity	ND	2.5–3.0	Probably only in fish with highest biomass gain	Martín‐Pérez et al. (2013)
TEF_bulk_	*Micropogonias undulatus* (Atlantic croaker)	Liver, muscle	Low quality: terrestrial sources (fish meal + plant‐based) Medium quality: terrestrial (fish meal + plant‐based) + marine sources (fish meal) Control feed: marine sources (fish meal)	No	Yes	2–4	Yes, only in fish with highest biomass gain	Mohan et al. (2016)
TEF_AA_	*Acanthopagrus butcheri* (Black bream)	Muscle	Fish meal feed Vegetable feed	Yes	Yes	0.9–1.2	No	Bloomfield et al. (2011)
TEF_AA_	*Thunnus orientalis* (Pacific bluefin tuna)	Muscle	70% sardine+ 21% squid+ 9% gelatin (N = non‐formulated diets)	No comparison, only one treatment	N/A	93.5	Yes^b^	Bradley et al. (2014)
TEF_AA_	*Carcarias taurus* (Tiger shark), *Negaprion brevirostris* (Lemon shark), *Triakis semifasciata* (Leopard shark), *Pristipomoides filamentosus* (Opakapaka)	Muscle	Non‐formulated diets: Anchovy, haddock, trevally, saithe, mackerel, whiting, mullet, octopus, krill, squid	No comparison, only one treatment	N/A	ND	ND	Hoen et al. (2014)
TEF_AA_	*Fundulus heteroclitus* (Mummichug)	Muscle	Plant‐based commercial fish pellet: wheat meal, soy meal, corn meal Omnivorous commercial fish pellet: fish meal, krill meal, wheat gluten, whey protein Clam Squid	Yes	Yes	2	ND	McMahon, Thorrold, et al. (2015)
TEF_AA_	*Seriola lalandi* (Pacific yellowtail)	Liver, muscle	Fish meal	No comparison, only one treatment	N/A	4	Yes	Barreto‐Curiel et al. (2017)

Notes

ND, no data; N/A, Not applicable.

a A WR = 3 (≈67% change in isotope turnover when assuming simple dilution conditions) was considered as a threshold for isotopic equilibrium.

b According to Madigan et al. (2012), during the experiment, sardines and squid were caught several times from the wild and may have varied in isotopic composition. Although fish increased in weight substantially, small variations in the isotopic composition of prey may have led to small biases in TEFs.

Furthermore, the use of artificial formulated fish feeds that do not consider nutrient requirements or that are not representative of the nutritional characteristics of natural diets consumed in the wild (such as the use of vegetable‐based diets to feed carnivorous fish) limits our ability to understand the sources of variability in TEF_AA_. Fish increase consumption rates to compensate for diets with low‐protein quality, and to meet both energy and essential nutrient demands for AAs, fatty acids and vitamins (e.g., Saravanan et al., 2012). This adjustment leads to an increase in the amount of dietary protein intake and catabolic activity that can ultimately increase isotope discrimination. From a nutritional perspective, the criteria for formulating or selecting diets and feeding regimes are key in feeding experiments designed to evaluate which dietary factors drive variability in TEFs.

Most studies on CSIA‐AA δ^15^N focusing on fish have analyzed a single tissue (mainly muscle) (e.g., Blanke et al., 2017; Bradley et al., 2015). Consequently, it is relatively unknown whether AA isotopic discrimination varies between different tissues for fish fed under the same dietary regime. Given that fish tissues can vary substantially in isotope turnover rates and reflect information for different feeding periods (Bradley et al., 2014; Herzka, 2005; Hesslein, Hallard, & Ramlal, 1993), analyzing more than one tissue from the same individuals can yield insights into switches in trophic level and feeding habits over different time scales (e.g., Kurle, 2009; Malpica‐Cruz, Herzka, Sosa‐Nishizaki, & Escobedo‐Olvera, 2013; McNeil, Drouillard, & Fisk, 2006). Muscle and liver metabolism are innately different and play specific functional roles. Muscle tissue is responsible for movement, while the liver is involved in assimilation processes, storage of glycogen and lipids, and excretion, as well as the metabolism of proteins and AA, carbohydrates, and lipids. The metabolism of the fish liver can adapt to variations in AA availability to meet energy and metabolic requirements (Kaushik & Seiliez, 2010); the same AA pool serves for both catabolic and anabolic processes (Cowey, 1975). Moreover, liver serves a regulatory function, adapting to nutrient fluxes in response to tissue and whole‐body requirements and the availability of dietary AAs (Enes, Panserat, Kaushik, & Oliva‐Teles, 2009). Isotope discrimination in AAs in muscle and liver tissues may therefore differ substantially, rendering the empirical determination of tissue‐specific TEFs necessary.

Fish fed high‐quality diets (with an adequate amino acid profile and high digestibility) assimilate and accrete as protein between 25% and 55% of the total AA in their diets (Cowey & Walton, 1989; Halver & Hardy, 2002; National Research Council, 2011). The rest of the dietary AA pool (45%–75%) is used to sustain metabolic processes, including maintenance AA requirements and inevitable AA catabolism. The former refers to the AA required to maintain the protein pools in equilibrium and has been estimated to comprise a small proportion of total AA requirements (5%–20%). The latter refers to AA catabolism that occurs even when enough energy for protein synthesis is provided (National Research Council, 2011). Thus, fish have inevitable catabolic processes that cannot be shut down. This inevitable AA catabolism is estimated to be between 20% and 40% of the digestible AAs consumed by the fish above maintenance requirement (National Research Council, 2011). While source and trophic AAs have been broadly characterized based on whether transamination (and the resulting isotope discrimination) occurs (e.g., Chikaraishi et al., 2009), deamination resulting from AA catabolism will also lead to isotope discrimination (see review by O'Connell, 2017). All AAs are subject to catabolic processes, and hence, the observed variation in both source and trophic TEF_AA_ can be at least partially attributed to AA catabolism.

Considering these facts, we evaluated independently the effect of protein quantity and quality on nitrogen TEF_bulk_ and TEF_AA_ for both liver and muscle tissues of the Pacific yellowtail (*Seriola lalandi*), a model carnivorous species. We assessed the relationship between TEF_bulk_ and TEF_AA_ and protein quantity and quality as a function of fish performance (growth rates, feed conversion ratios, protein efficiency rate, and protein productive value). We hypothesized that TEFs of source AAs would not differ among fish tissues equilibrated with diets differing in protein quantity and quality. For bulk tissue and trophic AAs, we hypothesized that TEFs would increase with increasing protein quantity, because fish should catabolize excess dietary protein resulting in higher excretion of ^15^N‐depleted nitrogen and decrease with increasing protein digestibility (quality) due to direct routing and assimilation of available protein into fish tissues, which involves limited catabolic processes.

## 2 METHODS

### 2.1 Experimental diets

We formulated five experimental diets to contain increasing levels of digestible protein (DP) by changing the quantity and quality of a single batch of high‐quality fish meal (that contain highly digestible protein and with an AA profile that meets nutritional requirements; Table 2 and Supporting Information Table S1). The main protein source was a high‐quality 60% crude protein (CP) content fishmeal (Special Select, Omega Protein, Texas, USA) made from menhaden that containing a reported 60% crude protein, 6% crude fat, 2% crude fiber, 4.3%–5.3% calcium, and 2.5% phosphorus. A review of the AA content reported in the Special Select fish meal relative to the AA‐specific dietary requirements of *S. lalandi* indicated that the diets had sufficient AA content to meet the species requirements (data not shown).

**Table 2:** Experimental diet design. Diet codes reflect the percentage of digestible plus non‐digestible crude protein in each diet

Diet code	Digestible crude protein (%)	Nondigestible crude protein (%)	Total protein (%)
40 + 0	40	0	40
50 + 0	50	0	50
60 + 0	60	0	60
40 + 10	40	10	50
50 + 10	50	10	60
Commercial	57	0	57

*Seriola lalandi* was used as a model for a carnivorous marine teleost species because it is easy to raise in captivity, its nutritional requirements are well characterized, and it exhibits very fast growth rates. Diets were formulated based on the known protein and AA requirements for *S. lalandi* (Masumoto, 2002; NRC, 2011). One had the optimal required protein level as described in those two references that are based on nutritional studies (50% CP), another one with lower protein level (40% CP) and a third one with higher protein level (60% CP; hereafter referred to as diets 40 + 0, 50 + 0, and 60 + 0, respectively). Two additional experimental diets were formulated to contain 50% and 60% total crude protein but with 40% and 50% estimated digestible protein, respectively. This was achieved by combining 10% non‐digestible protein with the 40% and 50% digestible protein for a total of 50% and 60% crude protein (hereafter 40 + 10 and 50 + 10 diets, respectively). The nondigestible protein was prepared using the fish meal treated with formaldehyde to reduce the digestibility of the protein source using the well‐known protocol described by Antoniewicz, van Vuuren, van der Koelen, and Kosmala (1992). This technique is commonly used in terrestrial animal (ruminants) nutrition studies to reduce protein digestibility (Wulf & Südekum, 2005), and has been successfully applied to fish nutrition studies (Durazo et al., 2010). Formaldehyde (FA) treatment of dietary protein sources is not harmful to experimental fish as indicated by high growth rates, and allows for the formulation of diets with the same protein source and amino acid profile but different digestible protein content.

Feed ingredients (Table 3) were ground to pass through a 1.02 mm diameter sieve. The ingredients were blended with the fish oil using a food mixer for 15 min, cold‐extruded with a meat grinder using a 3 mm die and air‐dried to a moisture content <10%. A commercially formulated diet for marine fish (Skretting, UK; ≥55% crude protein, ≥15% crude fat, ≥1% crude fiber, ≥11.4% ash) was used as reference to evaluate fish growth and nutritional performance (hereafter referred to as commercial diet).

**Table 3:** Formulation of the experimental diets (g ingredient/100 g diet) on dry weight basis and proximate analysis of the prepared diets and commercial reference diets. FA: formaldehyde

Ingredient (g/100 g diet)	Diet (40 + 0)	Diet (50 + 0)	Diet (60 + 0)	Diet (40 + 10)	Diet (50 + 10)
Casein	5	6.4	7.7	5	6.4
Fish meal^a^	50	64	77	50	64
Fish meal treated with FA	0	0	0	15.4	14.7
Jelly	3	3	3	3	3
Fish oil	17	12	8	14	8
Gelatinized starch	15	8	0.8	9.1	0.4
Cellulose	6.5	3.1	0	0	0
Vitamins	2	2	2	2	2
Mineral mix	1	1	1	1	1
Vitamin C	0.5	0.5	0.5	0.5	0.5
Total	100	100	100	100	100

Proximate composition	Diet (40 + 0)	Diet (50 + 0)	Diet (60 + 0)	Diet (40 + 10)	Diet (50 + 10)	Commercial diet
Total crude protein (%)	42.1 ± 0.2	51.9 ± 2.7	61.3 ± 1.6	49.5 ± 3.2	60.0 ± 0.2	56.9 ± 0.2
Lipids (%)	20.4 ± 0.5	16.1 ± 0.3	12.1 ± 1.0	10.1 ± 0.2	8.9 ± 1.7	9.0 ± 0.6
NFE (%)	19.6	14.2	8.6	14.3	7.2	
Ash (%)	16.8 ± 0.1	18.7 ± 0.1	21.5 ± 0.1	18.9 ± 0.5	21.5 ± 0.2	12.5 ± 0.2
Energy (kJ/g)	21.3	21.3	21.4	19.3	18.6	20.5
P:E (mg/kJ)	18.8	23.5	28.0	25.9	32.2	27.6

Notes

NFE, nitrogen‐free extract.

a Omega Protein high digestibility fish meal: 60% crude protein, 6% crude fat, 2% crude fiber, 4.3%–5.3% calcium, 2.5% phosphorus, <0.015% ethoxyquin.

Efficiency of the FA treatment was evaluated using a simple multienzyme pH‐STAT in vitro digestibility protein assay (Lazo, Holt, & Arnold, 2002). We consider the non‐FA‐treated fish meal as the digestible crude protein source and the FA‐treated fish meal as the non‐digestible crude protein (Table 2). Protein hydrolysis by commercial digestive enzymes was reduced by 91% in FA‐treated fish meal compared to non‐FA‐treated fish meal.

### 2.2 Animal culture and feeding

Juveniles were produced from eggs at a commercial Pacific yellowtail hatchery (Baja Seas, Baja California, Mexico). Early juveniles were brought to the Marine Fish Laboratory at the Center for Scientific Research and Higher Education of Ensenada (CICESE) and acclimated for 40 days in two 3 m^3^ raceways connected to a recirculating system. Juveniles were maintained at 20 ± 2°C, and salinity at 35 ± 1. Dissolved oxygen (DO) concentrations were kept above 6 mg/L and total ammonia [NH^3+^ NH^4+^] was lower than ≤1.0 mg/L. Raceways were cleaned twice a day and >70% of the water exchanged daily. Fish were hand‐fed four times a day using a feeding rate of 6% body weight per day (Nakada, 2000) with commercial diet containing: ≥57% crude protein, ≥15% crude fat, ≤0.2% crude fiber. Individual mortality was recorded daily.

Immediately before the experimental phase, juveniles *S. lalandi* were weighed to the nearest 0.1 g. We observed a bimodal size distribution, and therefore, fish were separated into two groups to minimize the initial variation in size and obtain precise relative weight gain estimates (Carleton & Martínez del Río, 2005). Fishes with an initial weight of 26 to 30 g (mean ± *SD*: 28 ± 2 g) were assigned to treatments 40 + 0, 50 + 0, and 60 + 0, and fishes with initial weights of 19 to 24 g (22 ± 2 g) to treatments 40 + 10, 50 + 10, and the commercial diet. Treatments were randomly allocated to tanks (*n* = 12 fish per tank, and *n* = 3 tanks per treatment), for a total of 216 individuals.

Each experimental tank had a recirculating water system coupled to a biological filter and a UV light lamp. Temperature, DO, food consumed, and mortality were recorded daily for each experimental tank. Juveniles were held near the optimal temperature for this species (22 ± 2°C) (Pirozzi & Booth, 2009). Other environmental conditions were maintained as described above.

Fish were fed a fixed amount based on the feeding rates suggested by Nakada (2000) for Pacific yellowtail. Feeding regimes were adjusted weekly based on the mean weight of the fish of each tank (range 5.5% body wt/day at the beginning to 2.4% body wt/day at the end of the trial). Feedings were fed three times a day for the first 26 days and twice a day thereafter. Weight (g) and standard length (SL; mm) of 5 individuals (randomly selected per tank) were measured weekly.

### 2.3 Sample collection

Ten fish were collected on day 0 for isotope and proximate analyses. Fish fed with treatments 40 + 0, 50 + 0, 60 + 0, and commercial diets were sampled four to five times throughout the experiment depending on the average relative increase in biomass (WR = weight_*t*_/weight_initial_) for each treatment. Fish in the 40 + 10 and 50 + 10 treatments were only sampled at the beginning and end of the experiment. WR was used to monitor growth because weight gain is a conservative estimate of the percent of isotopic turnover in juvenile fishes; isotopic equilibrium (a steady state between a consumer's isotope composition and its diet) to a new food source can be approached after a fourfold to sixfold increase in fish biomass (Herzka, 2005). Two fish were collected at ca. WR = 2, WR = 3, WR = 5, WR = 7 for isotope analysis of bulk tissue and individual amino acids during the experiment, and three fishes were collected at the end of the experiment. Fish were euthanized by placing them on ice, weighted and standard length (SL) measured before dorsal muscle and liver tissues were dissected. An additional individual from each tank was sacrificed for proximate analysis. Diet, muscle, and liver samples were frozen at −20°C pending isotope and proximate analyses.

### 2.4 Proximate analysis

Fish feeds, fish muscle, and liver tissues were analyzed for protein, lipid, ash, and nitrogen‐free extract. Liver was only analyzed for crude protein at the start of the experiment due to their small size. Crude protein content was estimated based on the percent nitrogen determined during bulk isotope analysis (see below) and calculated as % N × 6.25 (Jones, 1941). Lipid content and ash content were analyzed using the Folch method (Folch, Lees, & Stanley, 1956) and by incineration (Association of Official Analytical Chemists,A.O.A.C., 1990), respectively. Carbohydrate (including fiber) content was estimated as nitrogen‐free extract, or NFE (%) = 100 − % protein − % lipids − % ash. Dietary energy was estimated assuming 1 g protein = 5.6 kcal, 1 g lipid = 9.4 kcal, 1 g carbohydrate = 4.1 kcal (Webster & Lim, 2002). The P:E ratio was calculated for each diet.

### 2.5 Sample preparation for bulk isotope and CSIA‐AA analysis

Liver and muscle, diets, and the fish meal were thawed and dried at 60°C and ground into a powder. Lipids were not extracted from any of the samples to avoid bias associated with lipid extractions because several studies have documented a shift in δ^15^N values after lipid extractions in bulk tissues (Hesslein et al., 1993; Ingram et al., 2007; Pinnegar & Polunin, 1999; Ruiz‐Cooley, Garcia, & Hetherington, 2011). Lipid extraction may remove not only lipids but also lipoprotein compounds that have low δ^15^N values (Bodin, Le Loc'h, & Hily, 2007; Sotiropoulos, Tonn, & Wassenaar, 2004). Moreover, the variability of δ^15^N values may depend on the amount of fat, fatty acids, and lipoproteins of individuals that vary between tissues, and C:N ratios may not be a good predictor of lipid content (Ruiz‐Cooley et al., 2011).

For bulk isotope analysis, 0.8–1.2 mg of homogenized samples were weighed into tin capsules and sent to the Stable Isotope Facility of UC Davis. Fish feeds and samples were analyzed using an Elementar CUBE elemental analyzer (Elementar Analysensysteme GmbH, Langenselbold, Hessen, Germany) interfaced to a VisION isotope ratio mass spectrometer (IsoPrime, Stockport, U.K.). The standard deviations (*SD*) of the laboratory's quality assurance materials, bovine liver, nylon 5, and glutamic acid, were 0.1‰, 0.3‰, and 0.2‰ for δ^15^N, respectively. For CSIA‐AA, sample preparation involved acid hydrolysis of the fish feeds, fish muscle, and liver samples to liberate amino acids from proteins and subsequent derivatization by methyl chloroformate before sample injection into gas chromatograph (GC, protocol detailed in (Yarnes & Herszage, 2017) before analysis by gas chromatography/combustion/isotope ratio mass spectrometry (GC/C/IRMS). The δ^15^N values were determined by gas chromatography/combustion/isotope ratio mass spectrometry (GC/C/IRMS). CSIA of AAs was performed on a Thermo Trace Gas Chromatograph coupled to a Delta V Advantage IRMS via a GC IsoLink combustion interface (Thermo Electron, Bremen, Germany). During each measurement, provisional values were calculated by adjusting measured values to a coinjected internal reference material, l‐norleucine. Subsequently, an external reference mixture was used to calibrate each individual amino acid, such that the known δ^15^N value was obtained (Yarnes & Herszage, 2017). Each experimental sample was analyzed in duplicate. The use of alkyl chloroformates in the measurement of δ^15^N is relatively new, however, a recent comparison of δ^15^N‐AA measurements as methoxycarbonyl methyl esters (MOC; Walsh, He, & Yarnes, 2014) and N‐acetyl isopropyl esters (NAIP; Styring, Knowles, Fraser, Bogaard, & Evershed, 2012), a more traditional esterification‐acylation technique, yielded comparable δ^15^N‐AA results across a range of sample types (Yarnes & Herszage, 2017). The following amino acids were reproducibly quantified in all analyzed samples: Ala, Val, Gly, Ile, Leu, Pro, Asp, Phe, Glu, Lys, and Met. The *SD* was calculated from duplicate measurements on each liver and muscle sample and values are reported in the Supporting Information Table S1 (overall mean *SD*: 0.5‰ for liver and 0.4‰ for muscle; range *SD*: 0.2%–0.7‰ for liver and 0.2–0.6‰ for muscle). The SD of individual AAs from duplicates was generally below 0.8‰ for all AAs, except for Asp, Glu, and Lys in the diet samples only (1.0, 1.5, and 1.0‰, respectively). Accuracy of calibration and quality assurance mixtures was high, and the standard deviations of all AA standards were ≤1.2 ‰ (mean *SD*: 0.8‰; Supporting Information Table S2). Stable isotope values are expressed in standard delta notation (δ) with respect to atmospheric nitrogen: δ^15^N (‰) = ([*R*_sample_/*R*_standard_] − 1) × 10^3^, where *R* is the isotope ratio ^15^N:^14^N.

### 2.6 Growth performance and survival

Growth performance was assessed by calculating final body weight, absolute weight gain, specific growth rate (SGR; Halver & Hardy, 2002) and WR as a function of time. Nutritional response variables were calculated using the following formulas (De Silva & Anderson, 1995), where the initial weight (*W*_i_) and the weight at time *t* (*W*_*t*_) are in grams:

$$\text{Feed intake}\;(\text{g}\;{\text{fish}}^{-1}98\;{\text{day}}^{-1})=\text{sum}\;98\text{-day feed intake per fish}$$

Feed Conversion ratio (FCR)=feed intake(g)/fish weight gain(g)

Protein efficiency ratio(PER)=fish weight gain(g)/protein intake(g)

Protein productive value(PPV)=fish protein gain(g)/protein intake(g)

$$\begin{array}{cc} & \text{Survival}\;\left(\%\right)=100-\left[(\text{number of dead individuals}/\right.\\ & \left.\text{total individuals per tank})\times 100\right] \end{array}$$

Fish growth performance calculations using fish weight and body composition are expressed as dry weights and feed consumption rates are reported as wet weights.

### 2.7 Evaluation of isotopic equilibrium

To evaluate whether isotopic equilibrium was reached we first evaluated the pattern of isotopic turnover for two source (Phe and Gly) and two trophic (Glu and Ala) AAs. Phe and Glu were selected based on their widespread use and importance described in the literature. An asymptotic pattern is expected in the isotopic composition of liver and muscle tissue as a function of WR if isotopic equilibrium is reached. We also estimated the percent of isotopic turnover achieved in each treatment as a function of weight gain following Herzka (2005). These estimates are based on mass balance considerations that assume simple dilution conditions (i.e., growth is considered the only process driving isotopic turnover), and are thereby conservative. The WR for each treatment was also calculated and expressed relative to absolute weight. Because fish size differed between treatments on *d* = 0, percent isotopic turnover and WR were calculated separately for treatments with a mean initial weight of 22 and 28 g. The consistency between the final (δ^15^N_Final_) and prefinal (δ^15^N_Final‐1_) isotopic measurements in fish tissues was evaluated using an independent sample Student's *t* test.

### 2.8 Data and statistical analysis

Final measurements of tissue‐specific bulk δ^15^N values were calculated as TEF_bulk_ = δ^15^N_tissue_−δ^15^N_diet_. In the CSIA‐AA literature, TEF refers to the ^15^N enrichment with each AA with trophic level following Chikaraishi et al. (2015) and McMahon, Thorrold, et al. (2015):

$${\text{TEF}}_{\mathrm{AA}}=\delta^{15}{\text{N‐AA}}_{\mathrm{tissue}}-\delta^{15}{\text{N‐AA}}_{\mathrm{diet}}$$

where δ^15^N‐AA_tissue_ and δ^15^N‐AA_diet_ represent the nitrogen isotopic value each AA in the consumer's tissue and diet, respectively. Average values ±1*SD* of TEF_bulk_ and TEF_AA_ for each treatment were calculated based on individual δ^15^N‐AA values (*n* = 3) measured at the end of the experiment relative to the diets.

Statistical analyses were carried out using SYSTAT V 11. One‐way ANOVAs were used to test for differences in proximate composition, growth performance (WR, SGR), nutritional performance (FCR, PER, PPV) and survival between treatments. The effect of protein quantity and quality on final fish weight was tested with an ANCOVA using mean initial size as a covariate. Statistical analyses included the reference diet only when evaluating growth performance and nutritional response.

The absolute difference between TEF_AA_ for liver and muscle tissues were plotted for each amino acid and treatment. The effect of treatments on TEF_bulk_ and TEF_AA_ for liver and muscle were also tested with one‐way ANOVA. Assumptions of homogeneity of variances were checked using Levene's equal variance test. Tukey's honestly significantly different (HSD) test with *p* = 0.05 was applied to identify significant differences between treatment when ANOVA results indicated significant differences between treatments. To determine whether protein quantity influenced TEFs, we focused on post hoc test results comparing the 40 + 0, 50 + 0, and 60 + 0 treatments. To evaluate the effect of protein quality, we compared the 50 + 0 vs. 40 + 10 and the 50 + 10 vs. 60 + 0 treatments. The TEFs estimated for fish fed with the reference commercial diet were excluded from statistical analysis when evaluating the effect of protein quantity and quality because its quality varied in an uncontrolled fashion relative to our formulated experimental diets. Power analyses were run using a one‐way ANOVA model to estimate the probability of correctly rejecting the null hypothesis by setting an alpha level of 0.5 and *n* = 3. Student's *t* tests were applied to identify differences between liver and muscle tissue TEF_bulk_ and TEF_AA_ (alpha = 0.05).

## 3 RESULTS

### 3.1 Survival, growth, and nutritional response

There were no significant differences in mortality (*p* > 0.05, Table 4) among dietary treatments. Specific growth rates differed significantly among treatments (one‐way ANOVA, *df* = 5, *F* = 17.3, *p* < 0.001) and ranged from 1.3 to 2.1%/day. Growth rates differed significantly between protein levels, but did not differ significantly between treatments with same protein level but with different protein quality; 50 + 0 vs. 40 + 10 and 60 + 0 vs. 50 + 10 (Table 4). Final relative biomass gain (WR) ranged from 3.6 (40 + 0 diet) to 7.9 (commercial diet). The lowest WR value was found with the diet containing the lowest protein content. Final WR varied significantly between treatments with different protein content, but protein quality did not have a significant effect on final WR (Table 4).

**Table 4:** Growth performance and nutritional parameters of juvenile *Seriola lalandi* fed with diets differing in quantity and quality of digestible protein (DP) during a 98‐day feeding experiment (*n* = 3). Parameters: SGR = specific growth rate, WR = relative weight gain (*W*_*t*_/*W*_initial_), FCR = feed conversion rate, PER = protein efficiency rate, PPV = protein productive value. Values with different superscripts within a line are significantly different (*p* < 0.05) based on one‐way ANOVA followed by Tukey's HSD multiple comparison test. Diet codes indicate the percentage of digestible crude protein + nondigestible crude protein

	40 + 0 (mean ± *SD*)	50 + 0 (mean ± *SD*)	60 + 0 (mean ± *SD*)	40 + 10 (mean ± *SD*)	50 + 10 (mean ± *SD*)	Commercial (mean ± *SD*)
Initial body weight (g)	28.0 ± 2	28.0 ± 2	28.0 ± 2	21.5 ± 2	21.5 ± 2	21.5 ± 2
Final body weight (g)	100.1 ± 14.9^a^	153.4 ± 21.0^ab^	153.4 ± 11.8^b^	113.9 ± 2.9^a^	129.5 ± 15.2^a^	169.8 ± 6.3^b^
SGR (% body weight per day)	1.3 ± 0.2^a^	1.6 ± 0.2^ab^	1.7 ± 0.1^b^	1.7 ± 0.0^b^	1.8 ± 0.1^bc^	2.1 ± 0.0^c^
WR	3.6 ± 0.2^a^	5.5 ± 0.1^b^	5.5 ± 0.5^b^	5.3 ± 0.4^b^	6.0 ± 0.7^bc^	7.9 ± 0.9^c^
Feed intake (g 98 day^−1^ fish^−1^)	182.0 ± 4.4^a^	217.2 ± 0.7^b^	213.7 ± 5.0^b^	180.2 ± 4.2^a^	188.2 ± 2.3^a^	209.0 ± 3.8^b^
FCR	2.596 ± 0.5^a^	2.125 ± 0.5^ab^	1.712 ± 0.1^b^	1.953 ± 0.1^ab^	1.765 ± 0.3^ab^	1.411 ± 0.0^b^
PER	0.938 ± 0.2^ab^	0.900 ± 0.2^a^	0.939 ± 0.1^ab^	1.037 ± 0.1^ab^	0.953 ± 0.1^ab^	1.247 ± 0.0^b^
PPV	0.506 ± 0.1^ab^	0.465 ± 0.1^a^	0.488 ± 0.0^ab^	0.545 ± 0.1^ab^	0.526 ± 0.1^ab^	0.733 ± 0.2^b^
Survival (%)	89 ± 4.8^a^	75 ± 8.3^a^	84 ± 8.3^a^	81 ± 21.0^a^	81 ± 4.8^a^	81 ± 4.8^a^

Feed conversion ratios (FCR) ranged from 1.4 (commercial diet) to 2.6 (diet 40 + 0) (Table 4) and differed significantly among treatments (*F* = 5.3, *df* = 5, *p* = 0.008). The lowest (best) FCR value (1.4) was achieved by fish fed the commercial diet, followed by the 60 + 0 diet (1.7). Significant differences (one‐way ANOVA *F* = 5.3, *df* = 5, *p* = 0.036) were found in FCR among fish fed the higher protein quantity (60 + 0) treatment compared to the treatment with the lowest protein quantity (40 + 0). Treatments with different protein quality were not statistically significantly different in FCR. Protein efficiency ratios (PER) differed significantly among treatments (*F* = 3.3, *df* = 5, *p* = 0.04) and were lower in the higher protein and lower digestibility treatment. Protein productive values (PPV) differed significantly among treatments (*F* = 3.2, *df* = 5, *p* = 0.046). However, PPV did not differ between fish fed with diets varying in protein quantity and quality.

### 3.2 Proximate analysis

The protein content of initial liver tissue did not differ significantly between fish with initial mean weight of 28 and 22 g: Only lipid content in muscle tissue differed significantly (*p* = 0.05; Table 5). In liver tissue, the mean protein content of fish at the end of the experiment was variable but did not differ significantly among treatments (Table 5). There were no significant differences in protein, lipid, and ash content of muscle tissue at the end of the experiment among treatments (Table 5).

**Table 5:**
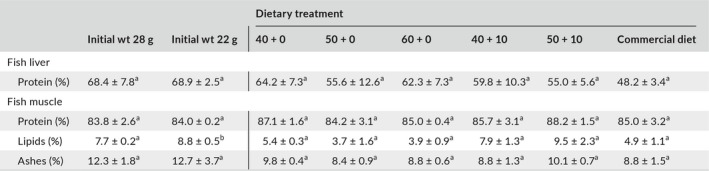
Proximate analysis of liver and muscle tissues (mean ± *SD*;* n* = 3 replicates per treatment) of juvenile *Seriola lalandi*. Fish with a mean weight of 28 and 22 g were fed diets differing in percentage and quality of digestible protein and sampled after a 98‐day feeding experiment. Proximate analyses are reported on dry weight basis. Percent ash and lipids could not be determined for liver tissue due to their small size. Diet codes indicate the sum of digestible protein + nondigestible protein. Different superscripts within a line are significantly different (*p* < 0.05) based on one‐way ANOVA followed by Tukey's HSD multiple comparison test

### 3.3 Evaluation of isotopic equilibrium

**Figure 1:**
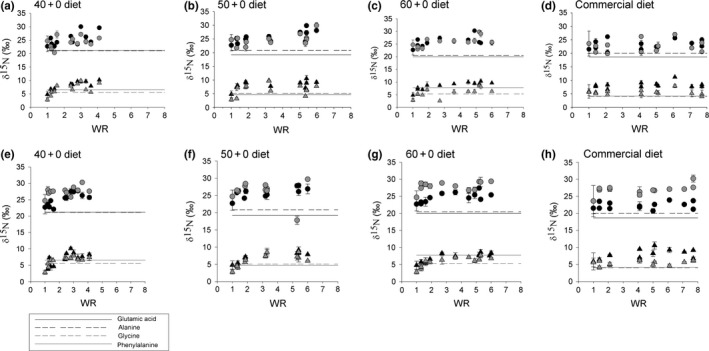
Pattern of nitrogen isotopic turnover of select amino acids in liver and muscle tissue of juvenile *Seriola lalandi* subjected to an abrupt dietary shift. Changes in isotopic ratios are expressed as a function of relative weight gain (WR = *W*_*t*_/*W*_initial_). δ^15^N_AA_ values are shown for liver (a–d) and muscle (e–h) tissues for two trophic amino acids (glutamic acid (black circles) and alanine (gray circles) and two source amino acids (phenylalanine in black triangles and glycine in gray triangles). Symbols represent individual fish; errors are 1 standard deviation of replicates for each sample. δ^15^N_AA_ of the diets are represented by horizontal lines. Diet codes indicate the percentage of digestible + nondigestible crude protein (see Table 2)

**Figure 2:**
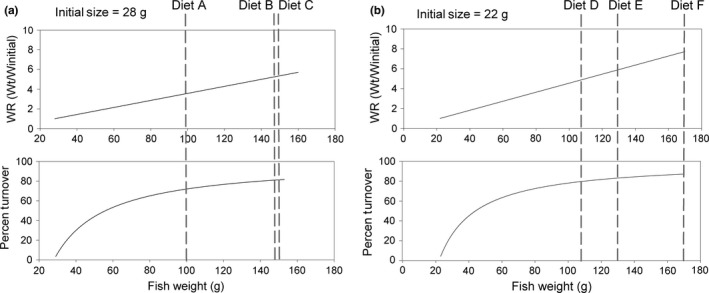
Simple dilution model of the expected isotope turnover pattern for juvenile *Seriola lalandi* subjected to dietary shift at a mean weight of 28 g (a) and 22 g (b). The mean relative weight gain (WR = *W*_*t*_/*W*_initial_) achieved by fish fed diets differing in the percentage of digestible + nondigestible crude protein is indicated by vertical lines, (diet A=40+0, diet B=50+0, diet C=60+0, diet D=50+10, diet E=60+10)

Isotopic shift patterns from the selected source‐ and trophic AAs exhibited an asymptotic behavior after the switch in diet (Figure 1). Isotopic equilibrium was approached at WR ≈ 3 by the four selected amino acids for all treatments and both tissues as well as the commercial diet. The calculated percent of isotopic turnover as a function of weight ranged from 72% to 87%. Fish with the slowest growth rate achieved a conservative estimate of isotopic turnover of 72% (Figure 2) at final WR = 3.6. The final (day 98) and next to last δ^15^N values from fish liver and muscle tissues did not differ significantly for bulk tissue (*t*‐student, *p* > 0.05) and the four selected AAs (*t*‐student, *p* > 0.05; Figure 1).

Isotopic equilibrium was therefore approached by the end of the experiment for all treatments in both fish tissues as indicated by three criteria: (a) the observed asymptotic isotopic pattern for the selected source‐ and trophic‐ AAs after an abrupt dietary shift, (b) the estimated high percent of isotopic turnover as a function of weight gain observed for all diets (>72%), and (c) the absence of statistical differences in the δ^15^N_AA_ between the last two sampling times for select AAs. Although we did not measure δ^15^N values during the course of the experiment for fish fed diets 40 + 10 and 50 + 10, we assume that equilibrium was also approached because fish achieved a greater WR than fish fed the lowest protein diet (i.e., diet 40 + 0). Also, WRs were similar to those calculated for fish fed diets 50 + 0 and 60 + 0. The rigorous confirmation of the approach to isotopic equilibrium is conducive to robust estimates of TEFs.

### 3.4 Isotope values of diets and final fish liver and muscle tissues

There was low variability in bulk δ^15^N values among the formulated diets (*SD* = 0.3‰), and fish liver and muscle tissues at the end of the experiment (Figure 4). Final individual δ^15^N values of source amino acids Phe, Lys, Met, and Gly ranged from 6.7 to 12.5‰ for liver, and from 7.6 to 10.9‰ for muscle. Final individual δ^15^N values of trophic amino acids Asp, Glu, Ile, Pro, Val, Leu, and Ala ranged from 21.2 to 26.8‰ for liver, and from 17.4 to 26.9‰ for muscle.

### 3.5 Bulk tissue TEFs

TEF_bulk_ for both liver and muscle tissues had limited variability among dietary treatments (Figure 5). In liver, TEF_bulk_ ranged from 2.1 ± 0.2‰ for the 40 + 10 diet to 2.8 ± 0.1‰ for the 50 + 10 diet. In fish fed the 40 + 10 diet, TEF_bulk_ was significantly lower compared to estimates for fish fed the other formulated feeds (*p* < 0.006, Table 6). In contrast, for muscle tissue, TEFs did not differ significantly (*p* = 0.45, Table 7) as a function of protein content and protein quality, ranging from 2.0 to 2.4‰.

**Table 6:** Mean ± *SD* of trophic enrichment factors (TEF) in bulk liver tissue and individual amino acids calculated for fish fed diets differing in protein quantity and quality. When a significant effect of diet was found with a one‐way ANOVA, (*p* < 0.05), Tukey's HSD multiple comparison tests were applied. Significant differences are indicated by superscript letters. Overall mean TEFs are reported when ANOVAs did not indicate differences between treatments. TEFs are expressed in ‰

	Treatment‐specific TEF (Percent crude protein + nondigestible crude protein)					TEF values (mean ± *SD*)	*F* ratio	*p*‐Value	Power analysis
	40 + 0	50 + 0	60 + 0	40 + 10	50 + 10				
Bulk liver	2.7 ± 0.1^a^	2.6 ± 0.3^a^	2.6 ± 0.2^a^	2.1 ± 0.2^b^	2.8 ± 0.1^a^		6.7	0.006	
Source AA									
Phe						2.3 ± 1.2	3.1	0.060	0.626
Lys	2.3 ± 1.4^a^	0.4 ± 0.6^ab^	−0.8 ± 0.7^ab^	−1.0 ± 1.1^ab^	−1.9 ± 2.5^b^		3.9	0.037	0.824
Met						2.5 ± 1.4	0.7	0.580	0.218
Gly						1.8 ± 1.5	0.5	0.700	0.152
Trophic AA									
Asp						4.2 ± 2.0	0.9	0.500	0.243
Glu						6.3 ± 2.2	0.3	0.850	0.109
Ile						4.0 ± 1.5	2.0	0.170	0.514
Pro						8.0 ± 1.3	1.6	0.260	0.42
Val						4.9 ± 1.5	0.2	0.900	0.087
Leu	5.4 ± 0.9^a^	4.8 ± 1.0^ab^	3.0 ± 1.4^b^	3.6 ± 0.5^ab^	3.5 ± 0.3^ab^		3.6	0.040	0.772
Ala						5.6 ± 2.4	1.9	0.170	0.457

**Table 7:** Mean ± *SD* of trophic enrichment factors (TEFs) for bulk muscle tissue and individual amino acids calculated for fish fed diets differing in protein quantity and quality. When a significant effect of diet was found with a one‐way ANOVA, (*p* < 0.05), Tukey's HSD multiple comparison tests were applied. Significant differences are indicated by superscript letters. Overall mean TEFs are reported when ANOVAs did not indicate differences between treatments. TEFs are expressed in ‰

	Treatment‐specific TEF (percent crude protein + nondigestible crude protein)					TEF values (mean ± *SD*)	*F* ratio	*p*‐Value	Power analysis
	40 + 0	50 + 0	60 + 0	40 + 10	50 + 10				
Bulk muscle						2.3 ± 0.3	1.0	0.450	
Source AA									
Phe	1.7 ± 0.6^a^	3.3 ± 0.3^c^	0.3 ± 0.5^ab^	−0.8 ± 0.6^b^	0.3 ± 0.4^ab^		20.3	0.000	1.000
Lys	0.4 ± 0.4^ac^	1.2 ± 0.1^a^	−1.0 ± 0.5^bc^	−0.1 ± 0.2^abc^	−1.8 ± 0.7^b^		9.3	0.004	1.000
Met	2.8 ± 0.8^a^	1.1 ± 1.6^ab^	2.0 ± 0.5^ab^	−0.3 ± 0.9^b^	0.5 ± 0.9^ab^		4.5	0.030	0.765
Gly						1.4 ± 0.8	0.46	0.760	0.125
Trophic AA									
Asp						2.9 ± 1.2	1.95	0.190	0.792
Glu	5.3 ± 0.9^ab^	8.1 ± 0.6^a^	5.6 ± 1.7^ab^	3.9 ± 0.6^ab^	3.1 ± 1.7^b^		5.0	0.020	0.981
Ile	5.5 ± 0.7^ac^	5.7 ± 0.3^ac^	6.1 ± 0.8^a^	2.0 ± 1.8^b^	3.4 ± 0.8^bc^		8.4	0.006	0.996
Pro						5.5 ± 1.1	2.7	0.100	0.621
Val						5.3 ± 1.1	3.2	0.070	0.668
Leu	6.6 ± 0.2^a^	5.6 ± 0.6^a^	5.4 ± 0.3^a^	3.1 ± 0.4^b^	4.1 ± 0.6^ab^		8.8	0.005	0.996
Ala						7.2 ± 1.7	2.25	0.150	0.756

### 3.6 Comparison between liver and muscle TEF_AA_

**Figure 3:**
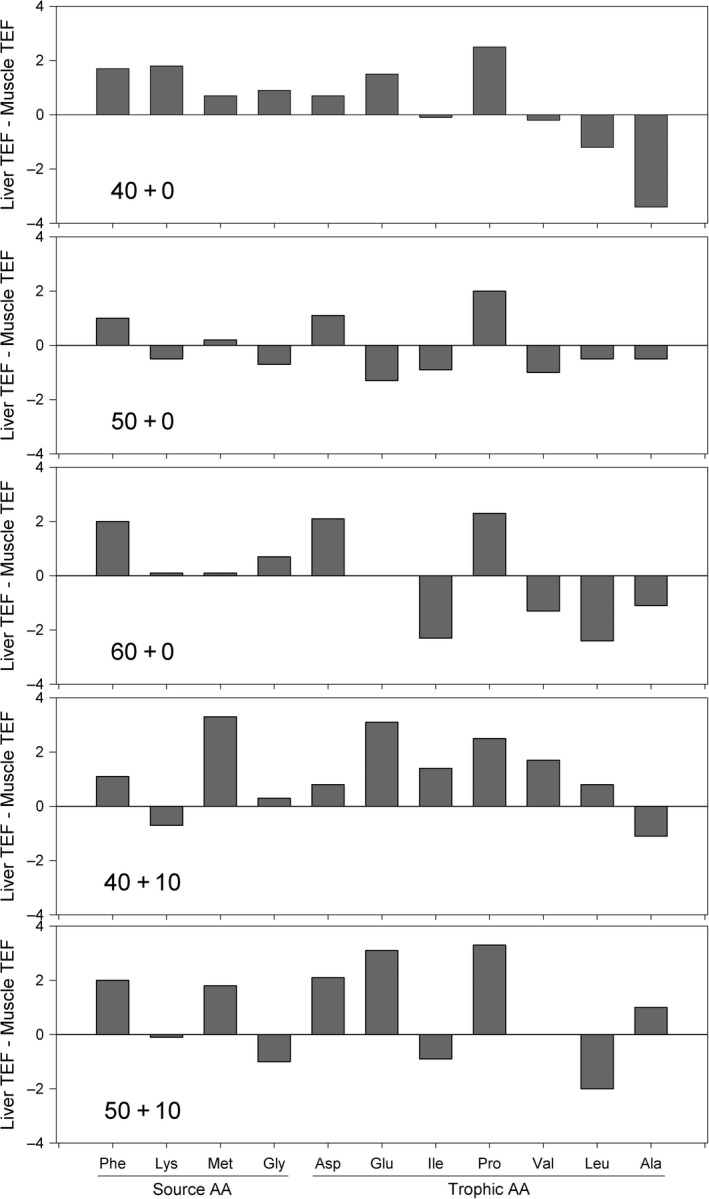
Difference between TEF for liver and muscle for each AA (Phe = phenylalanine, Lys = lysine, Met = methionine, Gly = glycine, Asp = aspartic acid, Glu = glutamic acid, Ile = isoleucine, Pro = proline, Val = valine, Leu = leucine, Ala = alanine) as a function of diets varying in protein quantity and quality. Dietary treatments are described in Table 2

There was generally a strong positive correlation between AA‐specific values between tissues (Supporting Information Figure S1). The strength of the association increased with protein content (*r* = 0.5 in the 40 + 0 to *r* = 0.8 in the 60 + 0 treatment). The difference in TEFs between tissues for each AA was inconsistent in magnitude and direction among treatments (Figure 3). In general, source AAs showed a low difference (<1‰) in TEFs between tissues in the optimal protein diet (50 + 0), whereas for the low‐protein quality diets (40 + 10 and 50 + 10), there were higher differences (up to 2‰). The difference in TEF_Phe_ was relatively consistent between tissues (1–2‰); Lys and Met had the lowest differences in the optimal and highest protein treatments (<1‰). TEF_Met_ varied little (<1‰) between treatments that did not include formalin‐treated fish meal, and showed higher discrimination (2–3.5‰) TEFs in the liver tissue of fish fed diets with decreased digestibility. The difference in TEF_Gly_ was low (<1‰) for all treatments. TEF_Lys_ had the highest difference between tissues in the diets with lowest protein content (40 + 0; ca. 2‰).

**Figure 4:**
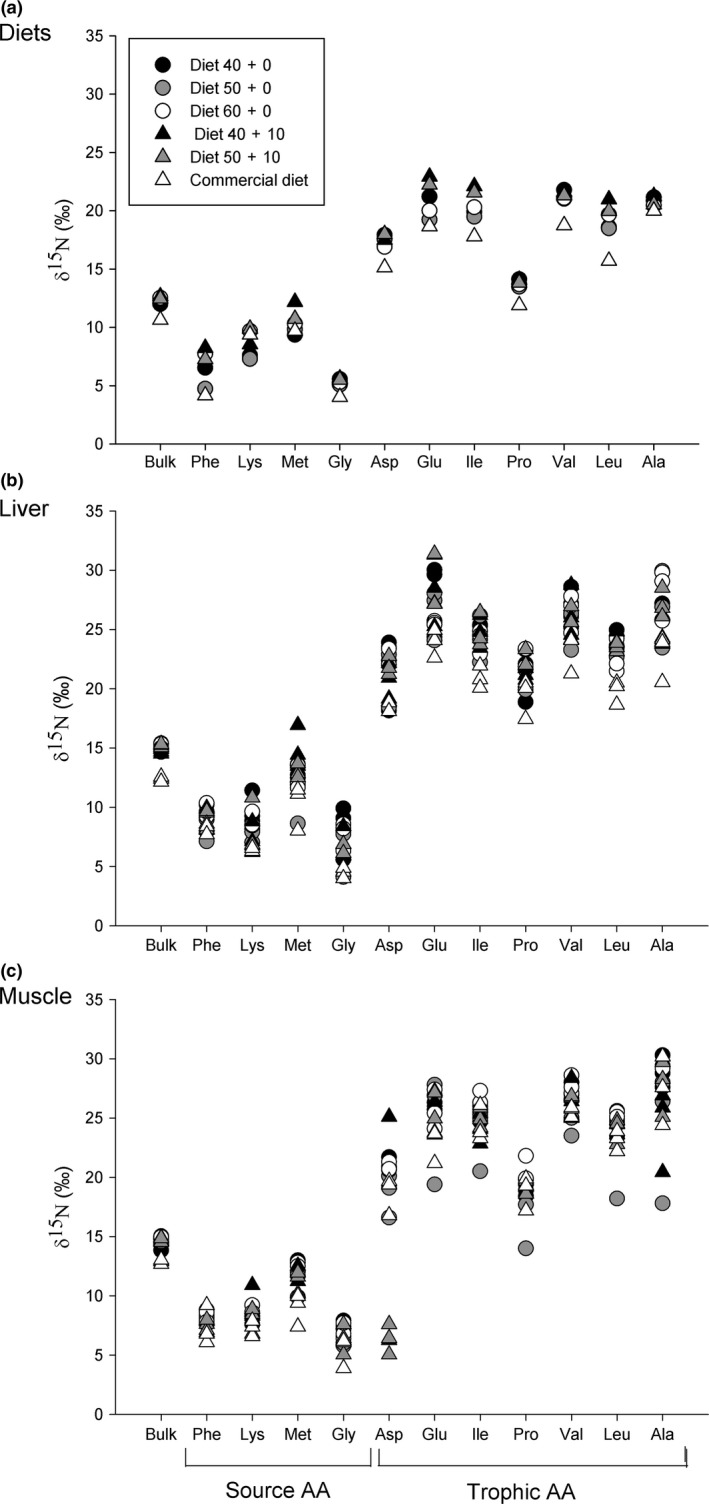
Bulk tissue and CSIA‐AA δ^15^N values of (a) experimental diets, (b) muscle, and (c) liver tissue (*n* = 3) of *S. lalandi* juveniles fed five formulated and one commercial diet for 98 d. Diets varied in the percentage of digestible crude protein (DP) + non‐digestible crude protein (NDP) as described in Table 1. Phe, phenylalanine; Lys, lysine; Met, methionine; Gly, glycine; Asp, aspartic acid; Glu, glutamic acid; Ile, isoleucine; Pro, proline; Val, valine; Leu, leucine; Ala, alanine. For simplicity, the error bars corresponding to the two measurements of isotopic composition performed in each sample are omitted

The difference in TEFs between liver and muscle tissues of trophic AAs varied substantially between treatments (Fig. 3). Nonetheless, fish fed the optimal protein diet had the lowest difference between tissues for all trophic AAs (less than 2‰). Pro had the highest TEFs in liver tissue, while Ala had the highest TEFs in muscle tissue. TEF_Glu_ had variable difference between tissues (up to 3.5‰) in all treatments except for the high‐protein diet. TEF_Ala_ had the lowest difference between tissues in the optimal protein (<1‰) and the highest in the low‐protein feed (almost 4‰). Proline was the only trophic AA with consistent and positive differences between liver and muscle tissues; liver tissue was more enriched in ^15^N. TEF_Val_ differed by <1‰ between tissues in the low‐protein treatments (40 + 0 and 50 + 10), and by 1–2‰ for the other treatments, and did not differ in the low‐protein digestibility treatment (50 + 10).

### 3.7 Amino acids TEF

**Figure 5:**
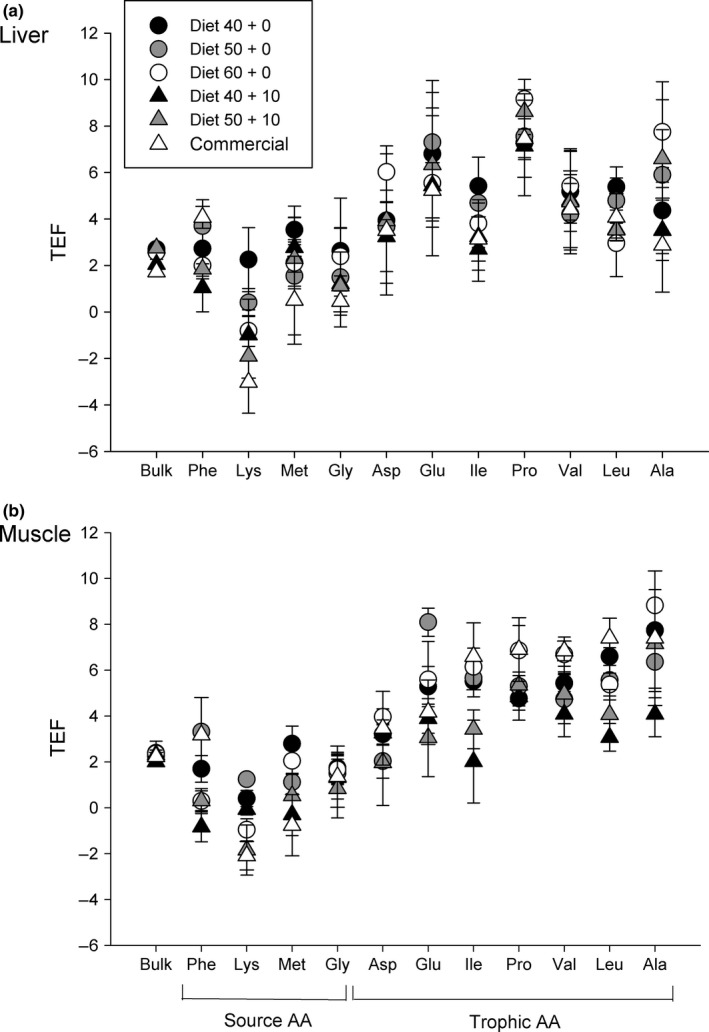
Trophic enrichment factors in bulk tissue (TEF_bulk_) and individual amino acids (TEF_AA_) for (a) liver and (b) muscle tissue (*n* = 3) of juvenile *Seriola lalandi* fed with five formulated feeds and one commercial diet differing in protein percentage and quality. Error bars represent the *SD* of TEFs for each dietary treatment. Diet codes indicate the percentage of digestible protein + non‐digestible protein

**Figure 6:**
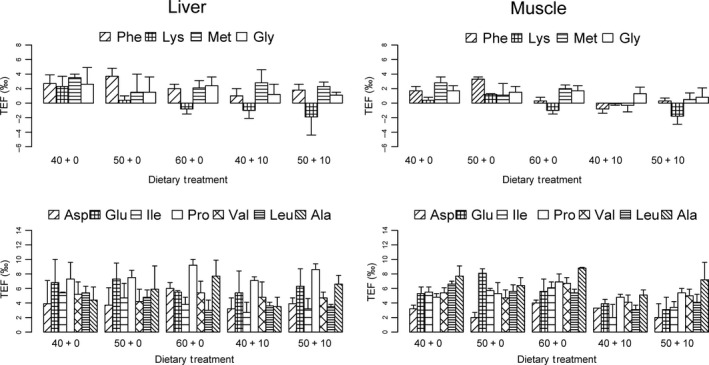
Trophic enrichment factors of individual amino acids (TEF_AA_) for liver (a) and (b) muscle tissue (*n* = 3) of juvenile *Seriola lalandi* fed with five formulated feeds differing in protein percentage and quality. Error bars represent the SD of TEF for each dietary treatment. Top panels: source AA. Bottom panels: trophic AA. Diet codes indicate the percentage of digestible protein + non‐digestible protein.

TEF_AA_ for source and trophic AAs were variable in liver and muscle tissues (Figures 5 and 6). For source AAs in liver, TEF_Lys_ exhibited significant differences among dietary treatments (*p* = 0.037, see Table 6), while TEFs for Phe, Met, and Gly did not differ significantly among treatments (Figure 6; Table 6). For muscle, the TEFs for Phe, Lys, and Met differed significantly among treatments (*p* < 0.001, *p* = 0.004, and p = 0.030, respectively); only TEF_Gly_ did not differ significantly among all treatments (Table 7).

Regarding TEFs for trophic AAs in liver tissue, TEF_Leu_ was the only one that differed significantly among treatments (*p* = 0.04; Table 6). In muscle tissue, TEFs Glu, Ile, and Leu values differ significantly among treatments (*p* = 0.020, *p* = 0.006, and *p* = 0.044, respectively). TEFs Asp, Pro, Val, and Ala did not differ significantly among treatments (Table 7).

## 4 DISCUSSION

The variable TEFs of all trophic AAs, and of some source AAs, indicate that isotopic discrimination varied between tissues depending on the dietary treatment. This may be related to the preferred energy sources used during fish growth, and the degree of transamination and deamination of specific AAs. The latter occurs due to AA catabolism; all AAs can be subject to catabolic processes in fish and other vertebrates (O'Connell, 2017). Below, we briefly discussed results of fish performance in relation to AA isotopic fractionation, and later, we discussed in detail the N isotopic fractionation for bulk tissues and AAs among and within each tissue.

### 4.1 Survival, growth, nutritional response

Dietary protein content had a significant effect on specific growth rate (SGR), and indicated significantly greater protein accretion in muscle tissue of fish fed the higher protein level diets compared with diet 40 + 0. Thus, our SGR values reflect adequate growth rates for this species reared under culture conditions irrespective of the presence or absence of treated fish meal. However, we observed a slightly higher SGR in fish fed diets with lower digestibility compared with those with the same crude protein level but higher digestibility. This result can be associated with the small initial fish size assigned to the 40 + 10 and 50 + 10 treatments.

Feed conversion rates (FCR) of fish fed experimental diets ranged from 1.7 to 2.6, which is within the range for *S. lalandi* (Moran, Pether, & Lee, 2009; O'Sullivan, 2005). Lower FCR were obtained in fish fed diets with higher protein content, reflecting better feed efficiency (Takakuwa, Fukada, Hosokawa, & Masumoto, 2006). The protein efficiency ratio (PER) and protein productive value (PPV) (that were estimated using total protein in the diets and assuming a decrease in digestibility of 100% in the fish meal treated with formalin), were not significantly different between fish fed diets differing in protein quantity and quality. However, calculating the PPV using the estimated available protein (i.e., the protein in the nontreated protein fish meal in the diet) results in a significant negative relationship (data not shown). This suggests that *S. lalandi*, like many other carnivorous fish, may have the ability to utilize dietary protein more efficiently when fed diets with lower protein quantity and/or lower quality (National Research Council, 2011). More efficient protein accretion should lead to lower TEFs, but we did not observe a clear relationship. Trophic AAs TEFs from the 40 + 0 and 40 + 10 diets were the lowest, especially for Asp TEF in both tissues. Fish fed the low‐protein diet (40 + 0) had the lowest growth rates and highest FCR, leading to limited AA catabolism and hence isotope discrimination.

The relationship between protein and energy in diets is important as lipids and carbohydrates can spare protein use as an energy source (i.e., protein sparing effect; National Research Council, 2011). The P:E ratios of our experimental diets ranged from 19.0 to 28.1 mg protein/kJ. The highest growth rates were obtained with a P:E of 23.8 (diet 50 + 0) and did not increase with higher P:E ratios. These results suggest that protein was in excess for diet 60 + 0, and the excess protein was probably burnt as energy. The highest trophic AAs TEFs in liver and muscle was found in the 60 + 0 diet. Fish possibly burned AAs as energy sources and reduced their protein efficiency when protein was in excess, which explain the high TEF_AA_ because fish likely metabolize more AAs.

### 4.2 Bulk tissue TEF as a function of protein quantity and quality

Despite the range of protein levels included in our formulated feeds, TEF_bulk_ did not vary as a function of protein quantity for either muscle or liver tissue. TEF bulk δ^15^N values were consistent with those previously reported for the same tissues in fish (McCutchan et al., 2003; Vanderklift & Ponsard, 2003) and about 1‰ lower than the 3.4‰ value typically used to calculate trophic level from fish muscle SIR.

Our results for TEF_bulk_ are inconsistent with the hypothesis proposed by Martínez del Río and Wolf (2005), and our hypothesis regarding the relationship between protein content and tissue TEFs, at least when considering a limited (albeit ecologically realistic) range of protein contents in the diets. The observed low variability in TEF_bulk_ from *S. lalandi* are also inconsistent with the results from previous studies that varied protein content without simultaneously influencing diet quality (particularly AA profiles) (see Table 1). For instance, Focken (2001) found a positive trend between whole fish TEF_bulk_ and feeding rate in Nile tilapia (*Oreochromis niloticus*) fed diets containing fish and wheat meals. However, there was not clear evidence that tissues reached isotopic equilibrium during the four‐week experiment as WRs were low (1.3–2.1). In contrast, the negative relationship between muscle TEF_bulk_ and protein content observed in gilthead sea bream (*Sparus aurata*) (Martín‐Pérez et al., 2013) may be a result of the result of an increased feed intake in fish fed the lower protein content diet, because fish were fed to satiation. This may explain the different relationship observed between protein content and TEF_bulk_ between their study and ours. The preferential assimilation of one of their protein sources may also contributed because different protein sources can drive N isotopic fractionation differently depending upon protein quality (Martín‐Pérez et al., 2013). Lastly, another study showed a positive relationship between muscle TEF_bulk_ and protein content in Nile tilapia fed a casein‐based diet fed at different levels, including in excess of the estimated maximal intake (Kelly & Martínez del Río, 2010). Higher TEF_bulk_ values were observed in diets with higher protein content, possibly due to high daily ration conducive to protein catabolism. A broader range of protein contents in the diets may therefore yield a positive relationship.

Protein quality (specifically protein digestibility) had a negligible effect on TEF_bulk_ of muscle tissue and a limited effect (0.5‰) in liver tissue. No previous studies report on the effect of protein digestibility on TEFs of fishes or other taxa. Other experiments evaluating the effect of varying the AA profile (another component of dietary quality) on TEF_bulk_ and avoiding a potentially confounding effect of covarying protein digestibility observed different results. Gaye‐Siessegger, Focken, Abel, and Becker (2007) evaluated AAs synthesis from their precursors relative to isotopic discrimination by raising Nile tilapia on three diets using fixed feeding rates. Whole fish TEF_bulk_ values were −0.3, 1.6, and 1.8‰, respectively, which are lower than our TEFs for liver and muscle. Their final WR values (1.1, 0.9, and 0.8, respectively) indicated low growth rates and weight loss; the authors concluded their results were likely due to the lack of absorption of synthetic AAs. Mohan et al. (2016) raised juvenile Atlantic croacker (*Micropogonias undulatus*) on diets considered of low (plant‐based, 32% protein) and medium (plant and animal‐based, 45% protein) quality in which the AA profiles necessarily differed. Their muscle TEF_bulk_ values were 6.5 and 4.7‰ for the low and medium protein quality, respectively, which are high relative to the values we obtained (2.3‰), possibly due to an imbalance in some AAs and the consequent metabolism of some NEAA. However, our results for liver TEF_bulk_ values are similar to the range these authors reported for the low and medium protein quality diets (3.0 and 2.1‰, respectively) and their high‐protein control diet (48% protein; 1.6‰).

### 4.3 Comparison between liver and muscle TEF_AA_

We found an increasing level of association between TEFs of liver and muscle in response to higher protein content (Supporting Information Figure S1). Fish fed diets with optimal or higher protein levels had more similar AA‐specific isotope enrichment factors. As dietary protein increased, the difference in the amino acid isotopic values between tissues decreased likely due to better feed efficiencies (lower FCE), which implies a lower amount of catabolism and hence lower isotope discrimination.

The differences in TEF_AA_ between liver and muscle support our hypothesis and agree with results from the few studies that estimated TEFs for multiple tissues at the intraspecific level. In harbor seals, Germain et al. (2013) found mean differences between blood serum and muscle of four individuals, ranging from 0.1 and 0.4‰ for Ala and Lys, to 5.9 and 6.7‰ for Gly and Ile. In fish, there is only one study that estimated TEFs for multiple tissues. Barreto‐Curiel, Focken, D'Abramo, and Viana (2017) fed *S. lalandi* a single diet with 43% protein content and found a difference of 3.3‰ for TEF_Met_ between liver and muscle, which is comparable to what we found for our low digestibility formulations. However, these authors calculated a difference of 3.4‰ between tissues for TEF_Phe_, and −0.7 and −0.9‰ for Lys and Gly, respectively, which differed from our results. Given that our study also used the same species, the differences in tissue‐specific TEFs between Barreto‐Curiel et al.'s (2017) and our study are possibly linked to differences in the quality of the protein sources, which includes the AA profiles, and the digestibility of the diets. Future studies should evaluate the effect of varying the dietary availability of specific AA on TEF estimates.

We hypothesized that source TEF_AA_ would have more consistent values between tissues than trophic AAs. Unexpectedly, TEF values of some source AAs varied by up to ca. 4‰ between tissues, and the difference was not consistent among dietary treatments (Fig. 3). TEF_Met_ differed by <1‰ between liver and muscle tissue in treatments varying protein quantity, and by up to 3.5‰ in fish fed diets with lower digestibility. Perhaps, the variable isotopic fractionation between tissues is related to the availability of Met in the diets: The lower availability of Met in the 40 + 10 diet might not have met the species’ dietary requirement, causing catabolism of endogenous Met in the liver.

We hypothesized that the TEFs of trophic AAs would exhibit a greater degree of difference between tissues than source AAs. Our results only partially agree with our hypothesis. The difference in TEF_Glu_ between liver and muscle tissue of fish fed diets of low‐protein quality was ca. 3‰, which is consistent with the 2.9‰ estimated by Barreto‐Curiel et al. (2017). The observed high differences in the TEFs of Glu between tissues for fish fed with low‐protein digestibility diets may be attributed to the dynamic and complex nature of Glu metabolism and its variability between both tissues, which is largely unknown in fishes (Li, Mai, Trushenski, & Wu, 2009). This NEAA plays numerous metabolic roles (Wu, 2009), and it is one of the preferred sources of metabolic energy in fishes. Its use as an energy source can be higher than glucose or fatty acids (Jia, Li, Zheng, & Wu, 2017). Higher isotope discrimination may depend on the degree in which Glu was used as an energy substrate or transaminated. All of these factors may underlie the observed high and variable isotopic discrimination in Glu between tissues and dietary protein attributes (i.e., quality and quantity) during *S. lalandi*'s growth.

In contrast to Glu, TEF_Pro_ showed consistent differences between muscle and liver TEFs for all dietary treatments. A consistent TEF_Pro_ was also detected in fish fed with diets that covaried protein quality and quantity (McMahon, Thorrold, et al., 2015), even in fish fed a plant‐based diet that possibly put fish under nutritional stress. Proline is synthesized from arginine (Arg) and glutamate/glutamine and is typically not considered an essential AA. Although ring closure of Glu is a pathway for Pro synthesis, arginine is also a major precursor via arginase; up to 40% of dietary Arg can be metabolized to form Pro, and glutamine and ornithine can be also be used as substrates (Wu et al., 2011). All these factors can lead to the observed differences in Pro and Glu TEFs.

Proline plays many important roles in protein synthesis and structure, metabolism and nutrition, as well as wound healing, antioxidative reactions, and immune responses (Wu et al., 2011). On a per‐gram basis, proline and hydroxyproline are the most abundant AAs in collagen; proline requirements for whole‐body protein synthesis are the highest among all AAs in fish (Li & Wu, 2018). Therefore, physiological needs for proline are particularly high. Although information about the role of proline is limited for fish, a study suggests that the liver probably synthesizes this AA to meet requirements, while muscle tissue may be more dependent upon the amount of proline available in the diet (Li et al., 2009). If true, this difference between tissues may explain the higher TEF_Pro_ in liver than muscle tissue.

A high difference TEF_Ile_ between tissues (>2‰) and higher TEFs in liver than in muscle was also observed by Barreto‐Curiel et al. (2017). The difference in TEF_Ile_ was higher in muscle tissue of fish fed the 60 + 0 diet with highest protein content (>2‰), suggesting higher catabolism in muscle and the consequent higher excretion of ^15^N‐depleted nitrogen. We observed a much higher TEF_Ala_ in muscle than liver tissue, which was also observed by Barreto‐Curiel et al. (2017).

In fish, most regulatory effects of nutrient utilization and metabolism initially occur in the liver, and its metabolism generates a cascade of events in other tissues (Enes et al., 2009). Liver tissue has a higher metabolic rate than muscle and it is where most of the NEAA are synthesized (Jürs & Bastrop, 1995), which may explain why the majority of AAs were more ^15^N‐enriched than in muscle tissue. Isotopic routing may also contribute to differences in TEFs between tissues, as nutrients are directed differentially to specific tissues (Tieszen & Fagre, 1993). Our results and the currently available literature to date nevertheless indicate that TEFs are tissue‐specific.

### 4.4 AA TEFs as a function of protein quantity

#### 4.4.1 Liver tissue

TEFs of Phe, Met, Lys, and Gly did not vary significantly with protein content among treatments, supporting our hypothesis. However, we did observe a marked trend toward a greater depletion in ^15^N in Lys TEFs with increasing protein content (TEF = 2.3 ± 1.4‰ to −0.8 ± 0.7‰ for diets 40 + 0 to 60 + 0, respectively), which is unexpected given its classification as a source AA. Barreto‐Curiel et al. (2017) also reported a negative TEFs for Lys (−0.7 ± 0.3‰). This may be related to differences in dietary lipid content, which was lower in the high‐protein diets (12.1 vs. 20.4‰ for the 40 + 0 vs. 60 + 0 diet, respectively), and 13.2‰ in the commercial diet of Barreto‐Curiel et al. (2017). Lys is used for the synthesis of carnitine, which is involved in the transport of long‐chain fatty acids into cells, and is often a limited AA in commercial fish diets, particularly those formulated with plant‐based protein sources (Li et al., 2009). Higher dietary lipid content would require more fatty acids transporters, which would increase Lys catabolism for the synthesis of carnitine, and would cause higher TEF_Lys_ in the low‐protein diet. Further studies are required to examine this possibility. Nevertheless, if Lys isotopic composition varies as a function of dietary lipid content, caution should be taken when interpreting its isotopic composition as a source AA in liver tissue.

Our mean TEF_Phe_ and TEF_Gly_ (2.3 ± 1.2‰ and 1.8 ± 1.5‰, respectively) are similar to those reported for the same species (3.2 ± 0.5‰ and 1.0 ± 0.4‰; Barreto‐Curiel et al., 2017), despite that Gly is now considered a “metabolic AA” due to its high variability in many taxa (O'Connell, 2017). TEF_Met_, however, differed by ca. 5‰ between our study (2.5 ± 1.4‰) and Barreto‐Curiel et al. (2017) (7.5 ± 1.7‰), possibly due to variations in Met, cysteine (Cys), and taurine (Tau) availability relative to dietary requirements. This is possible because Met is the first AA to be limiting in formulated feeds in fish, and being a sulfur AA, its metabolism is linked with that of Cys and Tau (Li et al., 2009). High TEFs for Met could be indicative of conversion to Cys, which involves the transmethylation–transsulfuration pathway and results in the cleave of the amino group, during which isotope discrimination could occur (O'Connell, 2017). Regardless of the mechanisms underlying the lack of differences in isotope discrimination, Phe, Gly, and Met in liver tissue did not vary with protein content and exhibit limited isotopic enrichment relative to the diets in liver tissue.

Trophic AAs in liver tissue had higher TEFs than those of source AAs, as expected (e.g., Bloomfield, Elsdon, Walther, Gier, & Gillanders, 2011; Chikaraishi et al., 2009; Hoen et al., 2014; McMahon, Thorrold, et al., 2015). In our study, proline exhibited the highest TEF (8.0 ± 1.3‰), followed by Glu (6.3 ± 2.2‰), Ala (5.6 ± 2.4‰), and Val (4.9 ± 1.5‰). This pattern differs from that of Barreto‐Curiel et al. (2017), who reported higher TEFs for Glu than Pro (8.4 ± 0.7‰ and 4.9 ± 0.8‰, respectively) and lower values for Ala (4.6 ± 0.88‰) and Val (4.1 ± 0.45‰). The differences in trophic TEFs values between these studies could be attributed to distinct dietary AA profiles and digestibility, and the consequent differential synthesis and catabolism of specific AAs.

We hypothesized an increase in TEF with increasing protein quantity for trophic AAs. However, our results lead us to reject this hypothesis for Asp, Glu, Ile, Pro, Val, and Ala because their TEFs did not differ between treatments. Despite the difference in dietary protein content, and the complexity of the metabolic pathways involved in the metabolism of these AAs (O'Connell, 2017), there were no differences in the level of isotope discrimination. In contrast to the rest of the trophic AAs, TEF_Leu_ showed a negative relationship with protein content, ranging from 5.4 ± 0.9‰ in the 40 + 0 diet to 3.0 ± 1.5‰ in the 60 + 0 diet. Previous studies also reported a negative relationship between dietary protein content and TEF_Leu_ in fish muscle (McMahon, Thorrold, et al., 2015). To our knowledge, there are no previous studies reporting data for fish liver tissue using a single protein source in experimental diets varying protein content. Although it has not been widely investigated in fish, leucine is considered a functional EAA (it plays a key role in determining the three‐dimensional structure of proteins and is thus involved in their functionality), and stimulates muscle protein synthesis in fish and mammals (Nakashima, Yakabe, Ishida, Yamazaki, & Abe, 2007; NRC, 2011). In our study, juvenile Pacific yellowtail grew adequately, but the treatment with the lower protein content exhibited lower growth rates and poorer food conversion efficiency, which could lead to more Leu catabolism (and hence higher isotope discrimination) for energy purposes than in the other treatments. However, it is important to consider that the catabolism of Leu is greater in tissues other than liver, like muscle, kidneys, and the central nervous system (NRC, 2011), and that Leu, Val, and Ile metabolism might be dependent in each other, which render the explanation of the differences in TEF_Leu_ difficult.

#### 4.4.2 Muscle tissue

Comparison between our TEF estimates and those of other studies can yield insight into the level of variation in isotope discrimination of AAs in fish muscle tissue. However, these studies covaried protein quantity and quality, and comparisons are necessarily qualitative when attempting to partition the contribution of protein quantity and quality to variation in AA‐specific TEFs. Unexpectedly, the TEFs of Phe and Lys showed significant differences among diets differing in protein content that lead us to reject our hypothesis for source AAs because they are not expected to vary as a function of protein content. These results challenge the current paradigm in which the CSIA‐AA of Phe and Lys in muscle tissue are assumed to reflect baseline isotope ratios.

TEF_Phe_ was significantly higher in the optimal protein diet (3.3‰), and the overall range of TEFs for Phe was also higher (0.3–3.3‰) than those reported for the omnivorous mummichug (*Fundulus heteroclitus*) fed diets differing in protein sources and quality (0.1–1.0‰; McMahon, Thorrold, et al., 2015). Blanke et al. (2017) also reported a limited range of TEF_Phe_ (−0.3 to 1.0‰) for four fish species fed a range of diets. Phe is an EAA whose metabolism is intimately related to that of Tyr via hydroxylation (Mathews & van Holde, 1996). In turn, Tyr can react with alpha‐keto‐glutarate, yielding p‐hydroxyphenylpyruvate and glutamate, which would imply deamination and consequently isotope discrimination (Mathews, 2007; O'Connell, 2017). Phe transamination with pyruvate can also occur, yielding Ala and phenylpyruvate, although this is thought to be a minor catabolic pathway (O'Connell, 2017). Phe has an important regulatory role in growth performance and Tyr is a precursor of neurotransmitters and hormones (Li et al., 2009). Thus, differences in Phe TEFs in diets differing in protein content and/or AA profile might be related to its specific functional and metabolic roles, and those of Tyr.

Similarly, TEF_Lys_ was the highest TEF (1.2‰) in fish fed the optimal protein diet, and the lowest TEF (−1.0‰) on the 60 + 0 diet. As Lys in muscle tissue is highly involved in the formation of collagen (Li et al., 2009; NRC, 2011), fish with higher growth rates should need to metabolize more Lys to support collagen production. However, we did not observe differences in growth rates between fish fed the 50 + 0 and 60 + 0 diets. Lys N can be transferred to the nitrogen pool through catabolic processes involving glutamate (O'Connell, 2017). Consequently, differences in the level of Lys catabolism between diets could lead to differences in TEFs.

In contrast, Met and Gly did not show significant differences in muscle tissue between diets differing in protein content, and both TEFs indicated limited discrimination (2.0‰ and 1.4‰, respectively). Barreto‐Curiel et al. (2017), however, reported a higher TEF_Met_ (4.5‰) for muscle tissue. As mentioned previously, Met is related to cysteine and taurine synthesis (Li et al., 2009), and as for other nontransaminating AAs, Met can be catabolized through deamination, which would lead to isotope discrimination and enrichment in the residual Met pool. The lack of differences in Met TEFs in muscle tissue therefore suggests a similar level of Met catabolism between diets.

As we mentioned before, the consistency in Gly TEFs was unexpected due to the high variability detected in several taxa of marine and freshwater consumers fed diets differing in protein sources (ca. 4‰; McMahon & McCarthy, 2016 and references therein), and its association with microbial degradation (McCarthy et al., 2007), and transamination. In fish, Gly metabolism is intimately linked with that of Cys; these two NEAA can be interconverted in the liver and kidneys and together they play a complex role in gluconeogenesis, sulfur AAs metabolism and the metabolism of fat (Li et al., 2009). McMahon, Thorrold, et al. (2015) reported Gly TEF values of −0.1 to 1.6‰ for an omnivorous fish, and Barreto‐Curiel et al. (2017) reported a value of 1.9‰ for muscle tissue of Pacific yellowtail. Taken together, these data and our results indicate Gly seems not to fractionate isotopically in N in response to changes in dietary protein content in marine fishes.

Despite that we hypothesized increasing TEF_AA_ values for trophic AAs with increasing protein quantity, trophic TEFs_AA_ varied but were not significantly different among 40 + 0, 50 + 0, and 60 + 0 diets and did not exhibit a specific pattern. These results disagree with previous findings in fish (McMahon, Thorrold, et al., 2015) in a study that covaried protein quantity and quality (Table 1). Their highest TEF values for trophic AAs were found in fish fed a plant‐based diet with a very‐low‐protein content. This plant‐based diet likely forced fish to catabolize their own body protein to meet energy requirements, leading to high isotope discrimination because, as we mentioned before, fish cannot metabolize carbohydrates efficiently and have high‐protein requirements (Booth, Moses, & Allan, 2013; Hemre, Mommsen, & Krogdahl, 2002). In the same study, Ala had the highest mean TEF (11.7‰) followed by Glu (10.8‰), while Pro had a more limited range (6.6–7.3‰) of values and the lowest TEFs among trophic AAs. Nevertheless, their Pro TEFs were somewhat higher than our mean Pro TEF value of 5.5‰. For *S. lalandi*, Barreto‐Curiel et al. (2017) reported higher TEFs for Pro (5.9‰) and than ours (5.5‰; Table 7), while lower TEFs for Ala (6.8‰) than ours (7.2‰) and relatively consistent TEF_Asp_ (3.7‰) with our TEF_Asp_ values (2.9‰). These inconsistencies in the trophic TEF_AA_ between our study and those of McMahon, Thorrold, et al. (2015) and Barreto‐Curiel et al. (2017) might be due to differences in protein sources and digestibility, as well as AA profiles.

### 4.5 TEFs as a function of protein quality

#### 4.5.1 Liver tissue

In liver tissue, the TEFs of source and trophic AAs did not differ between diets with decreased protein digestibility and hence quality. This is consistent with our hypothesis for source AAs. Liver tissue appears insensitive to variations in protein digestibility, at least within the protein levels and degree of reduced digestibility considered in our study. TEF_Lys_ did not differ significantly between treatments varying in protein quality; however, diets with low‐protein quality had negative TEF values, which was also reported by Barreto‐Curiel et al. (2017) and as was observed for liver tissue. As mentioned previously, dietary lipid levels may be intimately linked to Lys metabolism and consequently TEF values. Feeding studies with diets that only vary lipid content are required to examine the potential effect of lipid levels on TEF_Lys_.

#### 4.5.2 Muscle tissue

We hypothesized that the TEF_AA_ of source amino acids would not vary as a function of protein quality. However, in muscle tissue Phe exhibited a higher TEF (3.3‰) in the optimal diet (50 + 0) than in the lowest protein quality diet (−0.8‰ in diet 40 + 10). Notably, the fish fed the low‐protein diet that did not contain fish meal treated with formalin (diet 40 + 0) also had a significantly different TEF (1.7‰) than the 40 + 10 formulation. Comparison of our results with other studies indicates that TEF_Phe_ in fish muscle is variable. Barreto‐Curiel et al. (2017) reported a negative TEF_Phe_ (−0.16‰) for muscle of Pacific yellowtail. Bradley et al. (2014) and Hoen et al. (2014) reported low positive TEF_Phe_ values (1.5‰ in both studies) for Pacific bluefin tuna (*Thunnus orientalis*) and opakapaka, or pink snapper (*Pristipomoides filamentous*), respectively, which is similar to the TEFs of our fish fed the lowest protein content diet. This broad range of TEF_Phe_ values differs from the more limited range reported for fish fed diets differing in protein quantity that also varied in protein sources, and hence quality (0.1–1.0‰ in McMahon, Thorrold, et al., 2015; −0.3 to 1.0‰ in Blanke et al., 2017). Phe could reflect isotope discrimination when used directly as an energy substrate or when Tyr synthesized from Phe is catabolized, as the reactions involved include deamination (Mathews & van Holde, 1996; O'Connell, 2017). The differences in TEF_Phe_ between diets varying in protein digestibility may be attributed to variations in the extent to which this AA was used as an energy source or channeled for growth. Regardless of the cause, the studies available to date indicate that the isotopic composition of Phe in muscle tissue is sensitive to the nutritional characteristics of a fishes’ diet. More specifically, our results strongly indicate that isotope discrimination of Phe is sensitive to protein digestibility.

Although there were no significant differences in TEF_Lys_ between diets differing in protein quality, TEFs were negative in both treatments with decreased protein digestibility (−0.1 and −1.8‰) and TEFs showed a broad range of values for a source AA when considering all formulated feeds (from −1.8 to 1.7‰). Bradley et al. (2014) reported slightly negative TEF_Lys_ value (−0.3‰) for Pacific bluefin tuna and Hoen et al. (2014) reported positive values (ca. 0.5‰) for opakapaka; both studies held the fish in captivity and used wild‐caught prey as food sources. Barreto‐Curiel et al. (2017) also reported a low TEF_Lys_ in muscle (0.05‰), and McMahon, Polito, et al. (2015) and McMahon, Thorrold, et al. (2015) reported a positive range of TEF_Lys_ values (1.6–3.0‰). Thus, as with Phe, Lys TEFs of muscle do not appear to be consistent.

Similar to Lysine, Met TEFs did not show significant differences between protein quality in the diets, but the overall range of TEF_Met_ was broad for a source amino acid (ca. 3‰), and diets with decreased digestibility had lower TEFs (−0.3 and 0.5‰ for the 40 + 10 and 50 + 10 diets). Moreover, Met exhibited a significantly higher TEF (2.8‰) in the lowest protein content diet (40 + 0) than in the 40 + 10 diet (−0.3‰), which was formulated to have a similar digestible protein content. Barreto‐Curiel et al. (2017) also reported a high TEF_Met_ (4.2‰) for Pacific yellowtail. As we mentioned before, Met is an EAA that can be converted into cysteine and taurine (Li et al., 2009; Wu, 2009), and Met has also an important role as a precursor of other metabolic reactions and participates in the synthesis of glucose and glycogen (NRC, 2011). Differences in TEF_Met_ between dietary treatments may be due to the complexity of Met metabolism and the level of catabolism relative to its dietary availability and nutritional requirements.

Gly had a low mean TEF (1.4‰) in diets differing in protein digestibility. Once again, this consistency in Gly TEFs was unexpected because it has been reported to vary among several taxa of marine consumers that excrete ammonia (McMahon & McCarthy, 2016 and references therein), and may be the result of a limited range of protein levels within our experimental design. Bloomfield et al. (2011) reported TEF_Gly_ of −1.0‰ and 4.0‰ for black bream fish fed diets differing in protein sources. Bradley et al. (2014) reported slightly higher TEF_Gly_ value (3.4‰) than in our study, whereas Hoen et al. (2014) reported a wide range TEF_Gly_ values (from −7.0 to 5.0‰) for three elasmobranchs and one teleost; the enrichment factor for the teleost was 0.5‰. McMahon and McCarthy (2016) reported a low range TEF_Gly_ values (from −0.1 to 1.6‰) for muscle tissue of fish fed diets differing protein sources and quantity. Gly metabolism is linked to that of threonine (Thr) and Cys, and these three AAs can be catabolized through deamination through several pathways (O'Connell, 2017), which could lead to variation in isotope discrimination. Taken together, the studies available to date indicate that Gly TEFs vary in fish muscle tissue, although the underlying causes remain uncertain.

We hypothesized that the TEFs for trophic AAs would decrease with increasing protein digestibility; however, only TEF_Ile_ and TEF_Leu_ showed significant differences between the higher and lower quality diets. In both cases, TEFs were higher in the higher quality diets. The higher TEFs may reflect a greater degree of transamination or deamination in the diets with higher protein quality. Although our range of TEFs for Ile and Leu were similar to those reported for by Barreto‐Curiel et al. (2017) (4.9 and 5.1‰, respectively), previous studies have reported some higher TEFs for Ile (range: 5.2–9.4‰) and Leu (range 5.5–10.0‰) (McMahon, Thorrold, et al., 2015). Bloomfield et al. (2011) also reported very high TEF_Ile_ values and TEF_Leu_ values of fish fed fish meal (9.0 and 21.0‰) and vegetable‐based (9.5 and 20.1‰); these diets must have differed markedly in their AA profiles, and due to their limited growth, the fish may not have reached isotopic equilibrium (Table 1).

TEFs for Glu differed significantly between diets differing in protein digestibility, despite the relatively large level of variation between replicates in some treatments (maximum *SD* observed among replicates ≈1.7‰). TEFs for Glu spanned a large range of values (3.1–8.1‰), similarly to what was reported by McMahon, Thorrold et al. (2015) (5.6–10.8‰) and Blanke et al. (2017) (5.9–8.2‰). Bloomfield et al. (2011) reported higher TEF_Glu_ values (11.0 and 20.0‰), but as mentioned previously, fish may not have reached isotopic equilibrium and values may therefore be skewed. The TEFs for Glu reported by Bradley et al. (2014) (7.8‰), Hoen et al. (2014) (range 2.0–3.9‰), and Barreto‐Curiel et al. (2017) (5.5‰) also differ. Together, these results indicate that Glu in muscle varies substantially, even within the same taxa.

TEFs of the Asp, Pro, Val, Ala also did not differ significantly between diets differing protein quality, which reject our hypothesis. Among these AAs, Ala had the highest TEF value (7.2‰) and Asp the lowest (2.9‰). Bradley et al. (2014) reported relatively similar TEF_Ala_ (6.8‰), whereas Hoen et al. (2014) reported a wider range but lower TEF_Ala_ (ranged 0.5 to 6.0‰) and TEF_Asp_ (0.2 to 3.0‰). Barreto‐Curiel et al. (2017) reported high TEF_Ala_ (6.8‰) and a low TEF_Asp_ (3.7‰) for Pacific yellowtail. The lack of differences in TEFs may indicate that TEFs for Asp, Pro, Val, and Ala reflect the trophic step of a carnivorous fish. These results are unexpected given that diet quality represent one of the main current working hypothesis to explain the variability in many trophic AAs across trophic levels (TLs) including for the canonical trophic AA, Glu TEF, and TDF_Glu‐Phe_ (e.g., McMahon & McCarthy, 2016). For a high trophic level growing fish such as the carnivorous *S. lalandi*, the results of our study indicate that diet quality influence Glu TEFs, but does not have a significant effect on Asp, Pro, Val, and Ala TEFs. In particular, Asp TEF exhibited overall a relatively low isotope discrimination in muscle in response to diet quality but also quantity in comparison with other trophic AAs. These results suggest that Asp responds slightly to changes in dietary protein attributes.

## 5 SUMMARY AND RECOMMENDATIONS

In liver tissue, the TEFs of Phe, Met, Lys, and Gly did not vary with protein content and showed limited isotope fractionation relative to the diets. Only TEF_Lys_ decreased with protein content possibly in relation to higher dietary lipid content; further studies are required to examine this relationship. The low variability in TEFs of Asp, Glu, Ile, Pro, Val, and Ala with changes in protein content indicated that isotope discrimination remained relatively constant despite changes in dietary protein ranging from 40% to 60%, and only TEF_Leu_ decreased with higher protein content. In muscle, unexpectedly, Phe and Lys TEFs varied as a function of protein content despite that these AAs are believed to reflect baseline isotope ratios with minimum changes across trophic levels and diet compositions. Hence, careful consideration of whether these AAs are reflecting an isotopic baseline is warranted.

Regarding the effect of diet quality, we found that the TEFs of source and trophic AAs did not differ significantly between diets varying in protein digestibility in liver tissue. In muscle, the TEFs of Phe, Lys, and Met were sensitive to changes in protein quality, while Gly TEF exhibited low variability between treatments, indicating that Gly in muscle tissue may function as a robust source AA in teleosts, unlike other taxa for which a greater degree of variability has been observed (McMahon & McCarthy, 2016). Among trophic AAs, only TEFs of Glu, Ile, and Leu showed differences between diets differing protein digestibility. TEF_Glu_ exhibited a large range of values, which indicates that TEF_Glu_ varies substantially in teleost muscle in response to changes in protein quality.

Our results differ from the current paradigm that considers Phe to reflect baseline isotopic values because we found variable isotopic fractionation with differing diet content and protein quality in muscle (but not in liver tissue). Further, the observed variability in AAs TEFs between liver and muscle tissues indicates isotopic fractionation is variable between these tissues, and should not be assumed to be universal. In our study, the observed differences in TEF_AA_ between liver and muscle are likely driven by tissue‐specific functional roles and nutritional requirements relative to the availability of dietary AAs. Concurring with reviews of the premises underlying the application of stable isotope measurements in bulk tissues (Martínez del Río et al., 2009) and AAs (Ohkouchi et al., 2017), more experimental studies that consider AAs metabolism in response to dietary AA profiles and nutrient requirements are clearly needed for a better understanding of the causes underlying differences in TEFs between tissues. Our study highlights the need for carefully examining animal nutritional physiology before formulating diets, as well as independently evaluating the effect of dietary nutrients (e.g., protein quantity and quality, fatty acid, and carbohydrate content) in experimental feeding studies. Considering these aspects will help disentangle the variability in N isotopic fractionation in association with specific dietary protein attributes and will help us to identify the mechanisms that drive isotopic fractionation in bulk tissues and AAs.

## CONFLICT OF INTEREST

None declared.

## AUTHOR CONTRIBUTION

MTNP, SZH, and JPL designed the experiment. MTNP carried out the experiment. All authors participated in data analysis, interpretation, and elaboration of the manuscript.

## DATA ACCESSIBILITY

All of the raw data used to calculate trophic discrimination factors from bulk and CSIA measurements for each of the six experimental treatments are uploaded onto Dryad. We include fish weight for each of the six experimental treatments. DIO: PENDING.
