# Supplementary material for: Amino acid‐specific δ15N trophic enrichment factors in fish fed with formulated diets varying in protein quantity and quality
Source: Ecol Evol. 2018 Jul 30;8(18):9192–217. doi: 10.1002/ece3.4295 (PMC6194260; doi:10.1002/ece3.4295)
Supplement: Supplementary file 2 [file ECE3-8-9192-s002.docx]

Table S1. Average of the standard deviations calculated from the duplicate measurements of CSIA-AA made on each sample (n = 2) analyzed in this study.

| **Component** | **Diet** | **Liver** | **Muscle** |
| --- | --- | --- | --- |
| Alanine | 0.5 | 0.6 | 0.5 |
| Aspartic acid | 1.0 | 0.4 | 0.3 |
| Glutamic acid | 1.5 | 0.5 | 0.5 |
| Glycine | 0.5 | 0.5 | 0.4 |
| Isoleucine | 0.2 | 0.4 | 0.4 |
| Leucine | 0.3 | 0.2 | 0.3 |
| Lysine | 1.0 | 0.7 | 0.6 |
| Methionine | 0.8 | 0.6 | 0.4 |
| Phenylalanine | 0.7 | 0.4 | 0.5 |
| Proline | 0.3 | 0.3 | 0.2 |
| Valine | 0.6 | 0.5 | 0.3 |

Table S2. Precision and accuracy of nitrogen CSIA-AA. Average and standard deviations (SD) calculated from the two measurements of the laboratory´s internal standards: two mixtures of pure AA (n=79; UCD AA1 and UCD AA2), and two secondary quality assurance materials: a fish muscle and whale baleen (n=86). One mixture was used for isotopic calibration of measurements (UCD AA 1), while the other was not involved in corrections and served as the primary QA standard (UCD AA 2).

| **Component** | **UCD AA1**  **SD of** δ**^15^N** | **UCD AA1**  **Average of measured** δ**^15^N** | **Known** δ**^15^N for UCD AA1** | **UCD AA2**  **SD of** δ**^15^N** | **UCD AA2**  **Average of measured** δ**^15^N** | **Known** δ**^15^N for UCD AA2** | **MMS (Fish muscle)**  **SD of** δ**^15^N** | **RWB (whale baleen)**  **SD of** δ**^15^N** |
| --- | --- | --- | --- | --- | --- | --- | --- | --- |
| Ala | 0.9 | -6.82 | -6.72 | 1.2 | 40.65 | 41.40 | 1.4 | 1.3 |
| Asp | 0.5 | -2.32 | -2.34 | 0.7 | -2.51 | -2.29 | 1.1 | 0.6 |
| Glu | 0.7 | -4.24 | -4.17 | 1.2 | 47.79 | 47.60 | 1.2 | 1.0 |
| Gly | 0.7 | 0.81 | 0.82 | 1.1 | 0.93 | 0.73 | 1.0 | 1.0 |
| Ile | 0.7 | 2.39 | 2.53 | 0.7 | -3.79 | -3.53 | 1.2 | 1.2 |
| Leu | 0.4 | 9.21 | 9.24 | 0.6 | -5.07 | -4.29 | 1.0 | 0.9 |
| Lys | 0.9 | -0.92 | -1.36 | 1.1 | 0.14 | 0.47 | 1.5 | 1.3 |
| Met | 1.0 | -1.80 | -1.69 | n.m. | n.m. | n.m. | 1.1 | 1.1 |
| Phe | 0.5 | -1.24 | -1.14 | 0.7 | 1.53 | 2.06 | 1.2 | 1.1 |
| Pro | 0.4 | -1.51 | -1.44 | 0.7 | -4.93 | -4.11 | 0.8 | 0.6 |
| Val | 0.8 | 5.22 | 5.30 | 1.0 | -6.78 | -6.62 | 1.3 | 1.1 |

Fig S1. F Correlation of TEF_AA_ between liver and muscle tissues in diets varying in protein quantity. Symbols represent measurements of each AA in individual fish (Phe=phenylalanine, Lys=lysine, Met=methionine, Gly=glycine, Asp=aspartic acid, Glu=glutamic acid, Ile=isoleucine, Pro=proline, Val=valine, Leu=leucine, Ala=alanine). Dietary treatments are described in Table 2.
